# The Incidence Patterns Model to Estimate the Distribution of New HIV Infections in Sub-Saharan Africa: Development and Validation of a Mathematical Model

**DOI:** 10.1371/journal.pmed.1002121

**Published:** 2016-09-13

**Authors:** Annick Bórquez, Anne Cori, Erica L. Pufall, Jingo Kasule, Emma Slaymaker, Alison Price, Jocelyn Elmes, Basia Zaba, Amelia C. Crampin, Joseph Kagaayi, Tom Lutalo, Mark Urassa, Simon Gregson, Timothy B. Hallett

**Affiliations:** 1 Department of Infectious Disease Epidemiology, Imperial College London, London, United Kingdom; 2 Division of Global Public Health, University of California San Diego, San Diego, California, United States of America; 3 Rakai Health Sciences Program, Entebbe, Uganda; 4 Department of Population Health, London School of Hygiene &Tropical Medicine, London, United Kingdom; 5 Mwanza Research Centre, National Institute for Medical Research, Mwanza, Tanzania; University of Bern, SWITZERLAND

## Abstract

**Background:**

Programmatic planning in HIV requires estimates of the distribution of new HIV infections according to identifiable characteristics of individuals. In sub-Saharan Africa, robust routine data sources and historical epidemiological observations are available to inform and validate such estimates.

**Methods and Findings:**

We developed a predictive model, the Incidence Patterns Model (IPM), representing populations according to factors that have been demonstrated to be strongly associated with HIV acquisition risk: gender, marital/sexual activity status, geographic location, “key populations” based on risk behaviours (sex work, injecting drug use, and male-to-male sex), HIV and ART status within married or cohabiting unions, and circumcision status. The IPM estimates the distribution of new infections acquired by group based on these factors within a Bayesian framework accounting for regional prior information on demographic and epidemiological characteristics from trials or observational studies. We validated and trained the model against direct observations of HIV incidence by group in seven rounds of cohort data from four studies (“sites”) conducted in Manicaland, Zimbabwe; Rakai, Uganda; Karonga, Malawi; and Kisesa, Tanzania. The IPM performed well, with the projections’ credible intervals for the proportion of new infections per group overlapping the data’s confidence intervals for all groups in all rounds of data. In terms of geographical distribution, the projections’ credible intervals overlapped the confidence intervals for four out of seven rounds, which were used as proxies for administrative divisions in a country. We assessed model performance after internal training (within one site) and external training (between sites) by comparing mean posterior log-likelihoods and used the best model to estimate the distribution of HIV incidence in six countries (Gabon, Kenya, Malawi, Rwanda, Swaziland, and Zambia) in the region. We subsequently inferred the potential contribution of each group to transmission using a simple model that builds on the results from the IPM and makes further assumptions about sexual mixing patterns and transmission rates. In all countries except Swaziland, individuals in unions were the single group contributing to the largest proportion of new infections acquired (39%–77%), followed by never married women and men. Female sex workers accounted for a large proportion of new infections (5%–16%) compared to their population size. Individuals in unions were also the single largest contributor to the proportion of infections transmitted (35%–62%), followed by key populations and previously married men and women. Swaziland exhibited different incidence patterns, with never married men and women accounting for over 65% of new infections acquired and also contributing to a large proportion of infections transmitted (up to 56%). Between- and within-country variations indicated different incidence patterns in specific settings.

**Conclusions:**

It is possible to reliably predict the distribution of new HIV infections acquired using data routinely available in many countries in the sub-Saharan African region with a single relatively simple mathematical model. This tool would complement more specific analyses to guide resource allocation, data collection, and programme planning.

## Abstract 2

Annick Borquez and colleagues describe the development and validation of a mathematical model for estimating the distribution of new HIV infections in sub-Saharan Africa.

## Author Summary

**Why Was This Study Done?:**

- HIV national programmes require knowledge of incidence patterns across the country to tailor HIV prevention and testing programmes to the groups and places that will carry the incidence burden.
- Mathematical models provide an alternative to prospective cohort studies—which are costly and cannot be routinely implemented—to estimate HIV incidence.
- Sub-Saharan Africa (SSA) continues to bear the burden of the HIV epidemic and requires specifically tailored tools to inform HIV programmatic decision making.

**What Did the Researchers Do and Find?:**

- We developed the Incidence Patterns Model (IPM), which builds on strong data sources to produce geographically disaggregated estimates of the distribution of new infections in the next year by gender and marital/sexual activity status in SSA countries, taking into account the coverage of antiretroviral therapy and circumcision in these groups.
- We tested the IPM’s predictions on cohort data from four settings in the region for which the distribution of new infections is known and showed that the IPM was able to accurately estimate the distribution of new infections by group and geographical location.
- We applied the IPM to six countries in the region and identified broad differences in incidence patterns between and within countries and also illustrated plausible transmission patterns, providing insight into interventions to be prioritised.

**What Do These Findings Mean?:**

- The IPM is a useful tool to guide programmatic planning, identify data collection priorities, and support research and demonstration studies in particular groups.
- The IPM should be used in conjunction with contextual epidemiological studies, HIV programme and spending data, and models that provide a long-term perspective to develop a strategy that considers short- and long-term priorities.

## Introduction

Constraints in both health system capacity and financial resources drive countries toward programmatic efficiency in public health, including in their response to the HIV epidemic. To ensure that appropriate interventions and funds are allocated to the groups and settings that carry the largest incidence burden, a quantitative assessment of specific populations’ sizes and associated incidence patterns should be the foundation of the programmatic response [1]. In recent years, this response has shifted toward scaling up access to testing and treatment, as evidence supporting early treatment initiation to both improve health outcomes [2] and prevent transmission [3,4] accumulates. The new WHO treatment guidelines recommending antiretroviral therapy (ART) regardless of CD4 cell count and the UNAIDS 90-90-90 target—aiming to achieve knowledge of HIV status for 90% of those infected, treatment for 90% of those aware of their positive status, and viral suppression for 90% of those on treatment by 2020—reflect this commitment [5].

Understanding HIV incidence patterns allows identification of populations needing HIV services, including testing and prevention, and is therefore essential for reaching the treatment targets. Prospective cohort studies are considered the gold standard for estimating HIV incidence [6]; however, these are logistically complex and costly to implement, especially when estimates disaggregated by geographical location and population type are needed [6]. To circumvent these difficulties, alternative methods for calculating HIV incidence have been developed. Incidence assays, able to detect recent infection, are increasingly used to estimate incidence from cross-sectional data. Yet, their accuracy is compromised when ART biomarkers are unavailable and when stratifying the population by a range of factors, as sample sizes are reduced [6,7]. Further, several statistical and mathematical models have been developed to infer HIV incidence from HIV/AIDS case reports, HIV prevalence, and AIDS mortality data [8].

The UNAIDS Modes of Transmission (MoT) model is a tool that estimates the distribution of new infections in populations [9]. This simple model, used globally for over a decade [9], estimates the number of new infections in different groups according to their main mode of exposure, based on data from a variety sources on the average number of sexual or injecting contacts, condom use, and HIV prevalence among partners [10]. The MoT model’s Excel implementation makes it accessible to epidemiologists with no mathematical modelling background [10], and its estimates have been used to guide policy in several countries [11,12].

However, concerns have been raised regarding the reliability of the MoT model outputs in the sub-Saharan Africa (SSA) region [13,14]. First, the model structure assumes risk in the “low risk group” (defined as people who report being in a monogamous heterosexual relationship for the past 12 mo) to be homogeneous, while in practice this group includes sero-concordant positive couples, who cannot become newly infected, and sero-concordant negative couples, who can acquire infection only from external partnerships and are therefore not at risk. This can lead to overestimating the low risk group’s contribution to incidence and, by extension, to underestimating the incidence burden of key populations [13–15]. Second, the model includes groups that are not necessarily relevant from a programmatic perspective, whilst overlooking geographical heterogeneity, preventing the results from being used in practice for decision making [15]. Third, the model heavily relies on low quality or often unavailable behavioural data [14,15], leading to potentially inaccurate estimates. Finally, the uncertainty estimation method of the model is subjective, as the variation ranges of parameters are arbitrarily determined by the user.

To address these concerns, we propose a new generic approach for estimating the distribution of incident infections acquired according to identifiable determinants of risk in the generalised epidemics of SSA. The objectives of this study were to (1) describe the Incidence Patterns Model (IPM) for SSA, (2) validate and train the IPM using data from cohort studies in four countries, (3) apply the IPM to six different countries to show the predicted distributions of newly acquired HIV infections in different population groups, and (4) use the IPM outputs to infer the distributions of the groups transmitting HIV.

## Methods

Ethical approval was obtained from the Imperial College Research Ethics Committee (ICREC_9_3_13), the Biomedical Research and Training Institute’s institutional review board (AP91/10), and the Medical Research Council of Zimbabwe (MRCZ/A/681) for the Manicaland study; from the Malawi National Health Sciences Research Committee (#419) for the Karonga study; from the Uganda Virus Research Institute Research and Ethics Committee and the Uganda National Council for Science and Technology for the Rakai study; and from the Lake Zone Institutional Review Board and the Tanzania National Ethical Review Committee for the Kisesa study. Written informed consent was a requirement for participation in each of the four studies; thumbprint in front of a witness was required for participants who could not write.

In this section we describe the population structure, incidence inference methods, and statistical framework underpinning the IPM. We then present the model’s validation and training process, implemented using cohort data from ALPHA Network studies. The ALPHA Network is an initiative aiming to facilitate comparative and pooled analyses of data from ten community-based, longitudinal HIV studies in SSA that are based on a complete census of a geographical area and that use similar survey instruments [16,17]. Four of these studies had readily available data fit to the model requirements and were selected for the validation: the Manicaland, Karonga [18], Kisesa [19], and Rakai cohorts in Zimbabwe, Malawi, Tanzania, and Uganda, respectively. We describe the application of the trained model to six countries in the SSA region (Gabon, Kenya, Malawi, Rwanda, Swaziland, and Zambia) and define a simple model that uses the IPM outputs to infer the distribution of infections transmitted by different groups in these six countries.

### Incidence Patterns Model Structure and Statistical Framework

The IPM was developed to address limitations of the UNAIDS MoT model, and its principal advantages over the latter are described in detail throughout the Methods and summarised in Table 1. The IPM is better adapted to programmatic needs, offers a sounder methodological framework, and is validated against cohort data.

**Table 1:** Methodological comparison of the UNAIDS Modes of Transmission model and the Incidence Patterns Model.

Characteristic	MoT Model	SSA IPM
Group definitions	Based on sexual (and drug injecting) risk behaviours	Based on marital/sexual activity status or belonging to a key population
Geographical disaggregation	Not formally incorporated	Fundamental feature of the model
Incidence inference	Use of behavioural data to parameterise force of infection equation	Use of incidence estimates from direct observation in studies or inference from prevalence
Representation of risk in unions	Homogeneous	Explicit representation of sero-concordance/-discordance by sex, as well as circumcision and ART status
Uncertainty estimation method	Frequentist method allowing every parameter to vary within a range determined by the user (subjective)	Bayesian approach that incorporates accumulated information from the region and systematically accounts for sample size of data inputs
Data	Mix of sources including DHS	Formally based on DHS data to inform parameterisation
Validation	Not implemented	Implemented using data from four different settings in SSA

ART, antiretroviral therapy; DHS, Demographic and Health Surveys; IPM, Incidence Patterns Model; MoT, Modes of Transmission; SSA, sub-Saharan Africa.

### Stratification of the Population

In the model, the population is divided according to factors associated with HIV acquisition risk. The population is disaggregated by sex and marital/sexual activity status or membership in a key population, HIV and ART status for those in unions, and circumcision status as described in Table 2 and S1 Fig. The model is applied to each highest administrative division (here named “province”) in the country in order to account for geographical heterogeneity in HIV risk.

**Table 2:** Stratification of the population in the model by marital/sexual activity status, key population, circumcision status, and HIV and ART status within heterosexual unions; group definitions and HIV transmission routes considered.

Group	Marital/Sexual Activity Status or Key Population	HIV Status		Circumcision Status	ART Status	Definition	HIV Transmission
		**Man**	**Woman**				
**1**	**Sero-discordant unions**	**−**	**+**	Yes	No	Sero-discordant unions, sexually active in past 12 mo, where woman is HIV+ and not on ART and man is circumcised	Within partnership and external partnership transmission
**2**		**−**	**+**	Yes	Yes	Sero-discordant unions, sexually active in past 12 mo, where woman is HIV+ and on ART and man is circumcised	Same
**3**		**−**	**+**	No	No	Sero-discordant unions, sexually active in past 12 mo, where woman is HIV+ and not on ART and man is uncircumcised	Same
**4**		**−**	**+**	No	Yes	Sero-discordant unions, sexually active in past 12 mo, where woman is HIV+ and on ART and man is uncircumcised	Same
**5**		**+**	**−**		No	Sero-discordant unions, sexually active in past 12 mo, where man is HIV+ and not on ART	Same
**6**		**+**	**−**		Yes	Sero-discordant unions, sexually active in past 12 mo, where man is HIV+ and on ART	Same
**7**	**Sero-concordant HIV− unions**	**−**	**−**	Yes		Sero-concordant HIV− unions, sexually active in past 12 mo, where man circumcised	External partnership transmission only
**8**		**−**	**−**	No		Sero-concordant HIV− unions, sexually active in past 12 mo, where man uncircumcised	Same
**9**	**Sero-concordant HIV+ unions**	**+**	**+**			Sero-concordant HIV+ unions, sexually active in past 12 mo	No transmission
**10**	**Never married men**			Yes		Never married, circumcised men, sexually active in past 12 mo	External transmission (inferred from prevalence)
**11**				No		Never married, uncircumcised men, sexually active in past 12 mo	Same
**12**	**Never married women**					Never married women, sexually active in past 12 mo	Same
**13**	**Previously married men**			Yes		Widower/divorced/separated, circumcised men, sexually active in past 12 mo	Same
**14**				No		Widower/divorced/separated, uncircumcised men, sexually active in past 12 mo	Same
**15**	**Previously married women**					Widow/divorced/separated women, sexually active in past 12 mo	Same
**16**	**Not sexually active men**					Men not sexually active in past 12 mo	No transmission
**17**	**Not sexually active women**					Women not sexually active in past 12 mo	Same
**18**	**Female sex workers**					Female sex workers who sold sex regularly in the past 12 mo	External transmission (inferred from prevalence)
**19**	**Women who inject drugs**					Women who injected drugs in past 12 mo	Same
**20**	**Men who have sex with men**					Men who had sex with men in past 12 mo	Same
**21**	**Men who inject drugs**					Men who injected drugs in past 12 mo	Same

ART, antiretroviral therapy.

Marital status is strongly correlated with age but provides additional information on risk and is likely to be a better indicator to identify populations and deliver interventions. Married or cohabiting heterosexual unions are disaggregated according to sero-discordancy status and whether the man or the woman is HIV-positive, such that within partnership transmission is confined to sero-discordant unions. Polygamous unions are not explicitly represented in the model. However, the model allows for differences in the proportion of males and females in union.

The other marital/sexual activity status groups represented are never married men and women, previously married men and women (including widowed, divorced, and separated individuals), and men and women who were not sexually active in the past 12 mo (assumed to be at no risk of infection).

The key populations included are female sex workers (FSW), men who have sex with men (MSM) and people who inject drugs (PWID), disaggregated into men who inject drugs (MWID) and women who inject drugs (FWID).

### Incidence Estimation

The IPM is a single-time-step compartmental deterministic model. It builds on prior historical information on incidence patterns in the SSA region to estimate the distribution of new infections acquired in the next year. The methods are briefly described below, and further information is provided in S1 Text, section 1.1 to 1.3.

All men except MSM, MWID, and men who are not sexually active are stratified by circumcision status, as being circumcised reduces the risk of infection. Unions are disaggregated by ART status as HIV-positive individuals on ART have a reduced risk of onward transmission. The other groups are not disaggregated by ART status because the incidence calculation in these groups is not based on within- or between-group transmission estimates.

The calculation of the force of infection λ in each group *i* and province *r* is described below.

#### Unions

HIV incidence within unions is derived by directly applying infection hazards to the susceptible individuals corresponding to transmission from both stable (ξ_s_) and external (ξ_e_) partners in sero-discordant unions and from external partners only in sero-concordant HIV-negative unions. ϕ is the relative risk of infection among circumcised men and *о* is the relative risk of infection among people whose HIV-positive partner is on ART. *M*_1,*r*_ and *W*_1,*r*_ correspond to the number of men and women in union in region *r*, respectively. Groups 1 to 4 correspond to sero-discordant unions where the male partner is HIV-negative. These men are therefore exposed to transmission from their stable partners and from external partners. In groups 1 and 2, men are circumcised; in groups 2 and 4, women are on ART. Groups 5 and 6 correspond to sero-discordant unions where the female partner is HIV-negative. These groups are not disaggregated by circumcision status as it is assumed that circumcision does not reduce the risk of infection for the female partner; however, these groups are disaggregated by ART status, with men in group 6 receiving ART. Group 7 and 8 correspond to sero-concordant HIV-negative unions, in which individuals are exposed to infection only through external partners. Men in group 7 are circumcised, and the reduction in risk is adjusted to the proportion of men versus women in union. Within partnership transmission post-infection from an external contact in sero-concordant HIV-negative partnerships is not explicitly modelled but is taken into account in the external partnership hazard. Group 9 corresponds to sero-concordant HIV-positive unions, in which transmission results in no new infections.

λ1,r=ξs⋅ϕ+ξe⋅ϕλ2,r=ξs⋅ϕ⋅ο+ξe⋅ϕλ3,r=ξs+ξeλ4,r=ξs⋅ο+ξeλ5,r=ξs+ξeλ6,r=ξs⋅ο+ξeλ7,r=ξe⋅(ϕ⋅(M1,r(M1,r+W1,r))+(W1,r(M1,r+W1,r)))λ8,r=ξeλ9,r=0

#### Never married men and women

Incidence among never married men and women is estimated using measured prevalence (ρ_*i*,*r*_) and mean duration of sexual activity (δ_*i*,*r*_). It is based on the assumption that among young people, prevalence reflects incidence as a result of recent initiation of sexual activity and because prevalence is not yet influenced by mortality due to AIDS or ART treatment. Group 10 corresponds to circumcised, never married men, and therefore the force of infection is adjusted for the relative risk of circumcision ϕ.

$$\begin{array}{l} \lambda_{10,r}=\;\rho_{10,r}\cdot 1/\delta_{10,r}\cdot \phi \;\\ \lambda_{11,r}=\;\rho_{11,r}\cdot 1/\delta_{11,r}\\ \lambda_{12,r}=\;\rho_{12,r}\cdot 1/\delta_{12,r} \end{array}$$

#### Previously married men and women

Incidence among this population is calculated by applying the total infection hazard among unions (ξ_s_ + ξ_e_) multiplied by a relative risk (ς_*i*_) that is estimated from cohort studies with incidence data among both married and previously married participants. Group 13 corresponds to circumcised, previously married men, and therefore the force of infection is adjusted for the relative risk of circumcision ϕ.

$$\begin{array}{l} \lambda_{13,r}=\;\left(\xi_{\text{s}}+\;\xi_{\text{e}}\right)\cdot \varsigma_{13}\cdot \phi \\ \lambda_{14,r}=\;\left(\xi_{\text{s}}+\;\xi_{\text{e}}\right)\cdot \varsigma_{14}\\ \lambda_{15,r}=\;\left(\xi_{\text{s}}+\;\xi_{\text{e}}\right)\cdot \varsigma_{15} \end{array}$$

#### Key populations

Incidence among key populations is estimated by assuming it is a function of prevalence (ρ_*i*,*r*_) and turnover (δ_*i*,*r*_), which depends on the duration of infection, duration of risk practice, and life expectancy. The duration of infection accounts for the prevalence of ART in these populations.

$$\begin{array}{l} \lambda_{18,r}=\;\rho_{18,r}\cdot 1/\delta_{18,r}\\ \lambda_{19,r}=\;\rho_{19,r}\cdot 1/\delta_{19,r}\\ \lambda_{20,r}=\;\rho_{20,r}\cdot 1/\delta_{20,r}\\ \lambda_{21,r}=\;\rho_{21,r}\cdot 1/\delta_{21,r} \end{array}$$

### Estimation: Statistical Framework

A Bayesian framework is used to account for prior information on both demography and the HIV epidemic in the SSA region. We assume that countries in the region share basic demographic and epidemic patterns and that prior regional information will be useful in settings lacking local data to inform one of the model parameters.

### Data

One of the fundamental principles behind the IPM is its reliance on available data. The Demographic and Health Surveys (DHS) are extensive, high-quality household surveys carried out routinely every 5 y on average in the majority of SSA countries, providing data on a range of key topics, including HIV prevalence for men and women between the ages of 15 and 49 y representative of each administrative division [20,21]. The model requires province-level DHS data on the distribution of the population by group according to marital, sexual activity, and circumcision status as well as on the HIV prevalence by group and the duration of sexual activity among never married men and women. It also uses linked-unions data on the distribution of unions by sero-concordance and ART status (see S12 Table for a list of SSA countries with the data needed to apply the model).

Province-level data on the size of and HIV prevalence among key populations, as well as duration of exposure to risk practices, are also needed. This data can be obtained from Integrated Behavioural and Biological Surveys (IBBS) or from local research studies. Finally, the model requires data on the total number of new infections in the country in the past year, which can be estimated from DHS surveys using HIV incidence assays or taken directly from the latest UNAIDS estimates obtained from the Spectrum model [22]. It is preferable to use incidence data rather than model estimates, as the latter rely on both the quality of the inputs and of the structural assumptions.

### Specification of Prior Distributions

Prior distributions were specified for 38 parameters describing the size of the population and HIV prevalence in each marital/sexual activity status group, as well as the duration of sexual activity for never married men and women (see Table 3 for values). These prior distributions were obtained from recent DHS surveys in 19 countries in the region (S2 Table) that had included HIV testing. Pooled estimates for the SSA region as well as four sub-regions (western, eastern, central, and southern SSA) were calculated using weighted averages. The prior sample size was calculated by dividing the total sample size by 500 for the pooled SSA region and by a factor that produced a similar sample size for the sub-regions. This factor was determined based on average sample sizes when disaggregating the DHS data by province to give less weight to the priors when fitting to the data. Prior distributions for 11 parameters describing the size of the population and HIV prevalence as well as mean duration of risk practice in each key population were defined based on literature reviews. The prior sample sizes for the key population sizes and HIV prevalence were set at 500 for FSW, MWID, and FWID and at 250 for MSM to reflect higher variability in the last group.

**Table 3:** Priors for the sub-Saharan African Incidence Patterns Model to estimate the distribution of new infections acquired by key characteristic in the region.

Parameter Definition	Symbol	Distribution	Prior Mean					Sample Size					Ref.
			Pooled SSA	Eastern SSA	Central SSA	Western SSA	Southern SSA	Pooled SSA	Eastern SSA	Central SSA	Western SSA	Southern SSA	
**Population distribution: proportion of**													
Women not SA in past 12 mo	ν_w_	Beta	0.24	0.35	0.24	0.19	0.24	394	396	394	395	397	[21]
SA women who are in union	ω_w_	Beta	0.85	0.88	0.86	0.85	0.64	299	257	300	321	304	
SA women not in union who have never been married	ϒ_w_	Beta	0.74	0.59	0.55	0.84	0.81	46	33	45	46	108	
Men not SA in past 12 mo	ν_m_	Beta	0.26	0.35	0.24	0.19	0.29	184	234	215	117	236	
SA men who are in union	ω_m_	Beta	0.74	0.86	0.74	0.71	0.50	137	154	163	116	168	
SA men not in union who have never been married	ϒ_m_	Beta	0.92	0.88	0.89	0.95	0.94	37	26	44	34	83	
Never married men who are circ.	ϑ_n_	Beta	0.65	0.74	0.15	0.98	0.28	34	23	39	32	78	
Previously married men who are circ.	ϑ_p_	Beta	0.59	0.79	0.16	0.97	0.47	3	3	5	4	5	
Unions that are sero-concordant	Ө	Beta	0.95	0.98	0.90	0.97	0.83	76	103	82	66	47	
Sero-concordant unions that are HIV-negative	υ	Beta	0.97	0.99	0.91	0.99	0.72	73	99	74	64	39	
Sero-discordant unions where the man is HIV-negative	η	Beta	0.49	0.53	0.42	0.58	0.48	4	2	8	2	8	
Sero-concordant HIV-negative unions where the man is circ.	ϑ_c_	Beta	0.53	0.67	0.15	0.96	0.40	2	2	3	2	4	
Sero-discordant unions where the man is HIV-negative and circ.	ϑ_d_	Beta	0.57	0.68	0.21	0.98	0.41	79	98	67	63	29	
Women who are FSW	τ_FSW_	Beta	0.02	0.02	0.02	0.02	0.02	500	500	500	500	500	[23,24]
Women who are FWID	τ_FWID_	Beta	0.0004	0.0004	0.0004	0.0004	0.0004	500	500	500	500	500	[25]
Men who are MSM	τ_MSM_	Beta	0.02	0.02	0.02	0.02	0.02	250	250	250	250	250	[26]
Men who are MWID	τ_MWID_	Beta	0.0016	0.0016	0.0016	0.0016	0.0016	500	500	500	500	500	[27]
**HIV prevalence among**													
Men in union	ρ_cm_	Beta	0.06	0.02	0.15	0.02	0.32	473	121	100	83	74	[21]
Never married, circ. men	ρ_10_	Beta	0.02	0.01	0.04	0.01	0.10	101	15	5	29	22	
Never married, uncirc. men	ρ_11_	Beta	0.07	0.03	0.04	0.01	0.15	51	6	28	2	49	
Previously married, circ. men	ρ_13_	Beta	0.12	0.03	0.32	0.06	0.52	8	2	2	3	2	
Previously married, uncirc. men	ρ_14_	Beta	0.35	0.20	0.37	0.01	0.57	5	2	3	2	3	
Not SA men	ρ_16_	Beta	0.02	0.01	0.05	0.00	0.05	233	75	43	33	60	
Women in union	ρ_cw_	Beta	0.06	0.03	0.14	0.03	0.29	656	147	132	125	125	
Never married women	ρ_12_	Beta	0.12	0.05	0.14	0.03	0.34	96	10	16	20	64	
Previously married women	ρ_15_	Beta	0.27	0.12	0.39	0.11	0.61	33	2	12	3	13	
Not SA active women	ρ_17_	Beta	0.05	0.02	0.14	0.02	0.14	256	8	51	31	62	
FSW	ρ_18_	Beta	0.37	0.37	0.37	0.37	0.37	500	500	500	500	500	[28]
FWID	ρ_19_	Beta		0.13	0.13	0.13	0.13		500	500	500	500	[27,29]
MSM	ρ_20_	Beta		0.18	0.18	0.18	0.18		250	250	250	250	[29,30]
MWID	ρ_21_	Beta		0.13	0.13	0.13	0.13		500	500	500	500	[27,29]
**Biological parameters**													
Mean duration of SA among never married men	δ_10_, δ_11_	Log normal	5.49	5.46	5.59	5.97	5.46	0.87	0.62	1.12	1.06	1.26	[21]
Mean duration of SA among never married women	δ_12_	Log normal	4.74	3.02	4.84	5.11	5.72	2.07	3.50	0.86	1.37	0.46	
RR of infection among partners of people on ART	ο	Log normal	0.10	0.10	0.10	0.10	0.10	0.05	0.1	0.1	0.1	0.1	[3,4]
**RR of infection among circ. men**	**ψ**	**Log normal**	**0.40**	**0.34**	**0.34**	**0.34**	**0.34**	**0.02**	**0.005**	**0.005**	**0.005**	**0.005**	[31–33]
**Stable partnership transmission hazard**	**ξ**_**s**_	**Log normal**	**0.0175**	**0.0735**	**0.0735**	**0.0735**	**0.0735**	**2 × 10** ^**−5**^	**1 × 10** ^**−4**^	**1 × 10** ^**−4**^	**1 × 10** ^**−4**^	**1 × 10** ^**−4**^	[3,34,35]
**Casual partnership transmission hazard**	**ξ**_**e**_	**Log normal**	**0.007**	**0.0055**	**0.0055**	**0.0055**	**0.0055**	**3 × 10** ^**−6**^	**2 × 10** ^**−7**^	**2 × 10** ^**−7**^	**2 × 10** ^**−7**^	**2 × 10** ^**−7**^	
**RR of infection for previously versus currently married men**	**Ϛ**_**13**_ **, Ϛ**_**14**_	**Log normal**	**1.5**	**0.67**	**0.67**	**0.67**	**0.67**	**0.8**	**0.02**	**0.02**	**0.02**	**0.02**	[36,37]
**RR of infection for previously versus currently married women**	**Ϛ**_**15**_	**Log normal**	**2.22**	**0.92**	**0.92**	**0.92**	**0.92**	**0.9**	**0.08**	**0.08**	**0.08**	**0.08**	[37,38]
Mean duration of sex work among FSW	δ_18_	Log normal	4.38	4.38	4.38	4.38	4.38	0.5	0.5	0.5	0.5	0.5	[39]
Mean duration of SA among MSM	δ_20_	Log normal		10.00	10.00	10.00	10.00		2	2	2	2	
Mean duration of injecting among MWID and FWID	δ_19_, δ_21_	Log normal		4.31	4.31	4.31	4.31		10	10	10	10	
Mean ART coverage	ζ	Beta	0.37	0.37	0.37	0.37	0.37	100	100	100	100	100	[40]

The parameters in bold correspond to the incidence parameters trained on ALPHA Network cohort data. Parameters that share the same references are grouped in a box.

ART, antiretroviral therapy; circ., circumcised; FSW, female sex workers; FWID, women who inject drugs; MSM, men who have sex with men; MWID, men who inject drugs; RR, relative risk; SA, sexually active; SSA, sub-Saharan Africa; uncirc., uncircumcised.

Prior distributions were specified for six parameters determining the risk of infection in each of the population groups (infection hazards within and external to partnerships, relative risk of infection among circumcised compared to uncircumcised individuals, relative risk of infection among currently compared to previously married men and women, and ART coverage among unions) based on trials and cohort studies in the region (Table 3). These parameters are shared across provinces within a country.

### Parameter Estimation

**Fig 1:**
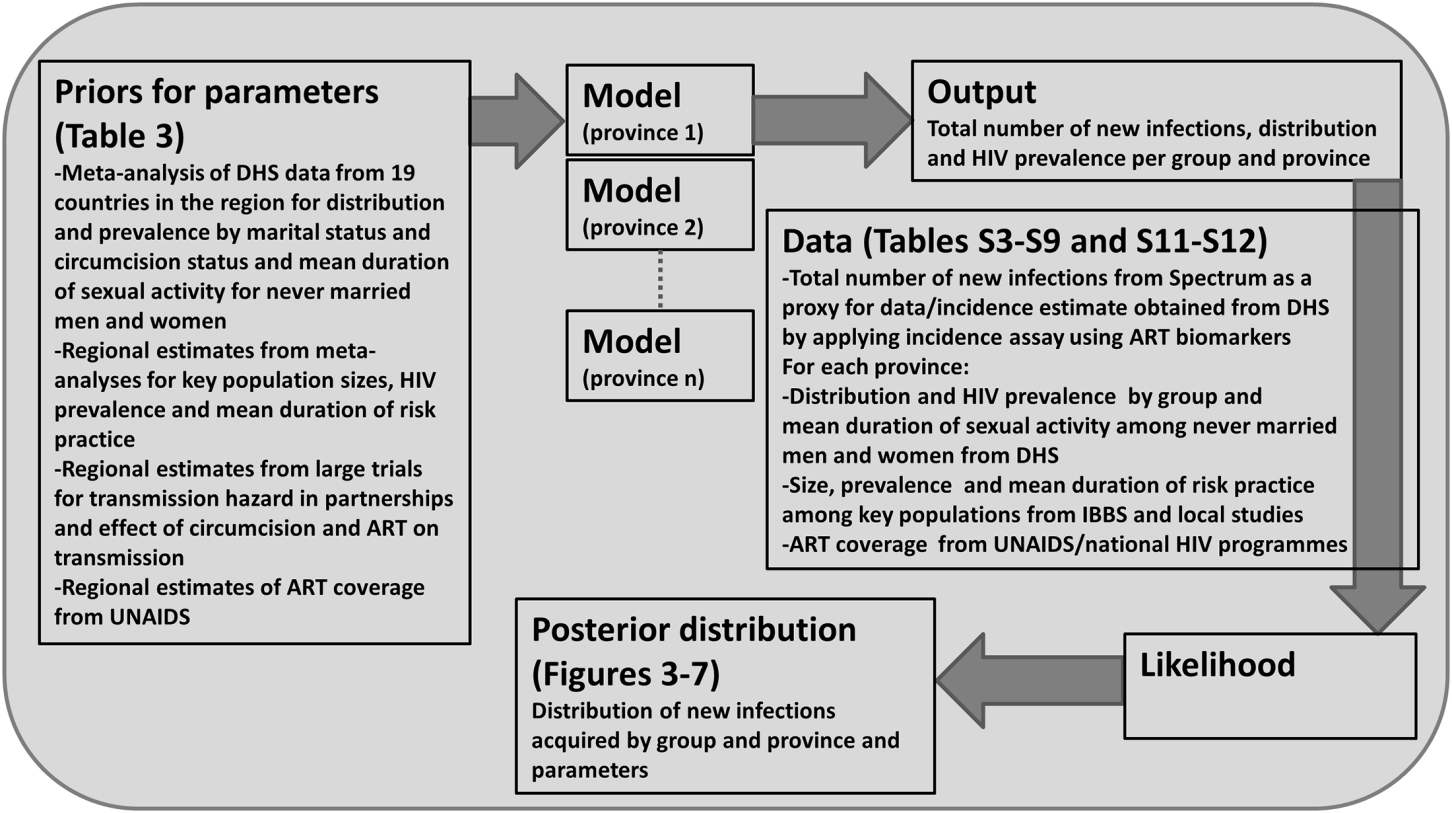
Bayesian statistical framework used to sample from the posterior distribution of the distribution of new infections by group. The model and MCMC algorithm were coded in Matlab version R2014a. ART, antiretroviral therapy; DHS, Demographic and Health Surveys.

A log-likelihood is calculated for each data point for the proportion of people in each group, the HIV prevalence in each group, the duration of sexual activity among never married men and women for each province, as well as for the total number of new infections predicted to occur in the next year. In some cases, data on the total number of new infections by province is available, and so a log-likelihood is calculated for each province. The total log-likelihood corresponds to the sum of all log-likelihoods listed. We used a Markov chain Monte Carlo (MCMC) approach to sample from the posterior distribution and report the mean and 2.5 and 97.5 percentiles as the parameter estimate and 95% credible interval, respectively (details are provided in S1 Text, section 1.3). The place of each component within the Bayesian framework is summarised in Fig 1.

### Validation and Training of the Incidence Patterns Model

To evaluate the model’s performance in different epidemic settings, it was applied to cohort data from four studies in the ALPHA Network. The extent to which the model could be improved by “training” it with local historical data was also investigated. Pairs of serial sero-surveillance surveys, referred to as “rounds” of data, were used, with one round available from Karonga (Malawi; surveys 1 to 3 of the original study), one round from Kisesa (Tanzania; surveys 5 to 6 of the original study), two rounds from Manicaland (Zimbabwe; surveys 3 to 4 and 4 to 5 of the original study), and three rounds from Rakai (Uganda; surveys 11 to 12, 12 to 13, and 13 to 14 of the original study), to assess whether (1) the projected distribution of new infections by group accurately represented the observed distribution (validation); (2) the projected geographical distribution of new infections accurately represented the observed distribution when using the different rounds of data as proxies for administrative divisions of one country (validation); (3) the model’s performance was improved when training it on historical data (“internal training”); the proposed training approach was to fit the model to data on the number of new infections in each group, as opposed to the total number of new infections, for one round of data, and use the corresponding posterior distributions of the incidence parameters as priors when applying the model on the next round of data; (4) the model’s performance was improved when training it on data from another site with similar HIV prevalence patterns (“external training”); and (5) the model’s performance was improved when training it on data from all other sites (“global training”).

The model’s performance using internal training was assessed on data from round 4 of Manicaland (using priors informed by round 3) and on data from rounds 12 and 13 of Rakai (using priors informed by rounds 11 and 12, respectively). The model’s performance using external training was assessed on each round of Rakai, using priors informed by all the rounds of Manicaland, and vice versa. This external training pairing was also implemented for Karonga and Kisesa. The model’s performance using global training was assessed on all rounds of data from all sites, using priors informed simultaneously by all rounds of all other sites.

Data to inform the model were extracted for each round of each of the four cohorts using STATA version MP13. No data were available on male-to-male sex or injecting drug use, and so the model could not be validated for these populations. The Manicaland survey coupled with the Women at Risk survey [41] provided data on paid sex that allowed validating the model for FSW.

Further details on the cohorts, results from the statistical analyses, and methods for the validation and training are provided in S1 Text, section 2.2 and 2.3, and S3–S9 Tables.

### Estimation of HIV Acquisition

After validating and training the IPM on data from the ALPHA Network studies, we applied the model to data from Gabon, Kenya, Malawi, Rwanda, Swaziland, and Zambia (S10 and S11 Tables). Basic demographic and epidemiological information on the six countries is provided in Table 4.

**Table 4:** Descriptive information on demographic and HIV patterns for the six countries studied.

Characteristic	Rwanda	Gabon	Zambia	Kenya	Malawi	Swaziland
SSA sub-region	Eastern	Western	Central	Eastern	Central	Southern
Population size	10,516,000	1,751,000	13,047,000	46,760,000	13,096,000	1,200,000
Proportion age 15–49 y	48%	49%	46%	47%	46%	51%
Number of provinces	5	10	9	8	3	4
Country HIV prevalence	3.0%	4.1%	13.3%	6.4%	10.7%	25.9%
ART coverage	65%	45%	32%	42%	36%	38%

ART, antiretroviral therapy; SSA, sub-Saharan Africa.

### Estimation of HIV Transmission

**Fig 2:**
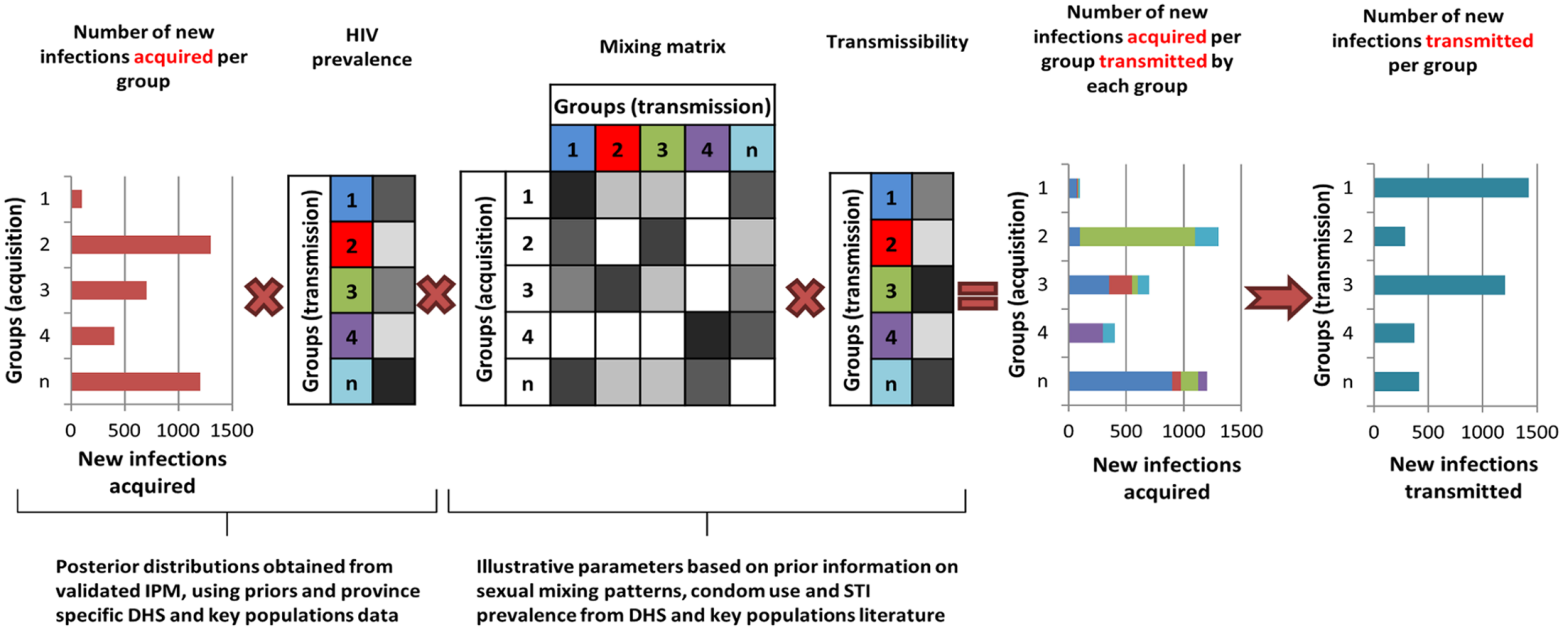
The transmission model based on the Incidence Patterns Model. The transmission model uses the posterior distribution from the IPM on the number of new infections acquired and the HIV prevalence in each population group to estimate the distribution of infections transmitted by each group using prior information on the mixing patterns between groups, transmissibility (depending on transmission probability, condom use, STI prevalence), and ART coverage in each group. The diagram is illustrative and does not specifically represent the groups described in the study. The intensity of the grey cells reflects the magnitude of the factors described. DHS, Demographic and Health Surveys; IPM, Incidence Patterns Model; STI, sexually transmitted infection.

In order to provide an indication of the plausible differences in the contribution of each population group to HIV acquisition and transmission, the outputs from the IPM were used to derive the proportion of transmission events attributable to each group in these six countries. It is important to consider transmission as an output, as some groups will account for a small proportion of new infections acquired as a result of their small population size but may contribute substantially to transmission if, for instance, the individuals in these groups have a high number of sexual partners and low levels of condom use. For the purpose of this study, a simple model was implemented to allocate new infections acquired in each group as estimated by the IPM to the different groups using assumptions on the sexual mixing patterns between groups and on the relative transmissibility from each group (S1 Text, section 4; S13 Table). This is illustrated in Fig 2. A prior distribution for the mixing matrix, corresponding to the proportion of all sexual contacts in a group that happen with individuals in each of the other groups, was defined based on available information from the DHS on sexual mixing patterns by age, on reporting of extramarital sex among men and women in union, and on paying for sex among men by age. Similarly, we assumed a prior distribution for the relative transmissibility of a group, corresponding to the mean cumulative probability of transmitting HIV to partners over the course of a year, based on factors that are known to influence the risk of transmission including HIV transmission probabilities per sex act for men and women, relative risk of transmission through anal sex and sharing injecting equipment compared to vaginal sex, average condom use, and sexually transmitted infection (STI) prevalence (S1 Text, section 4; S13 Table). New infections attributable to within partnership transmission in unions can be directly obtained from the IPM (as it explicitly represents within partnership and external partnership transmission hazards) and were accounted for in the derivations.

## Results

### Validation and Training

**Fig 3:**
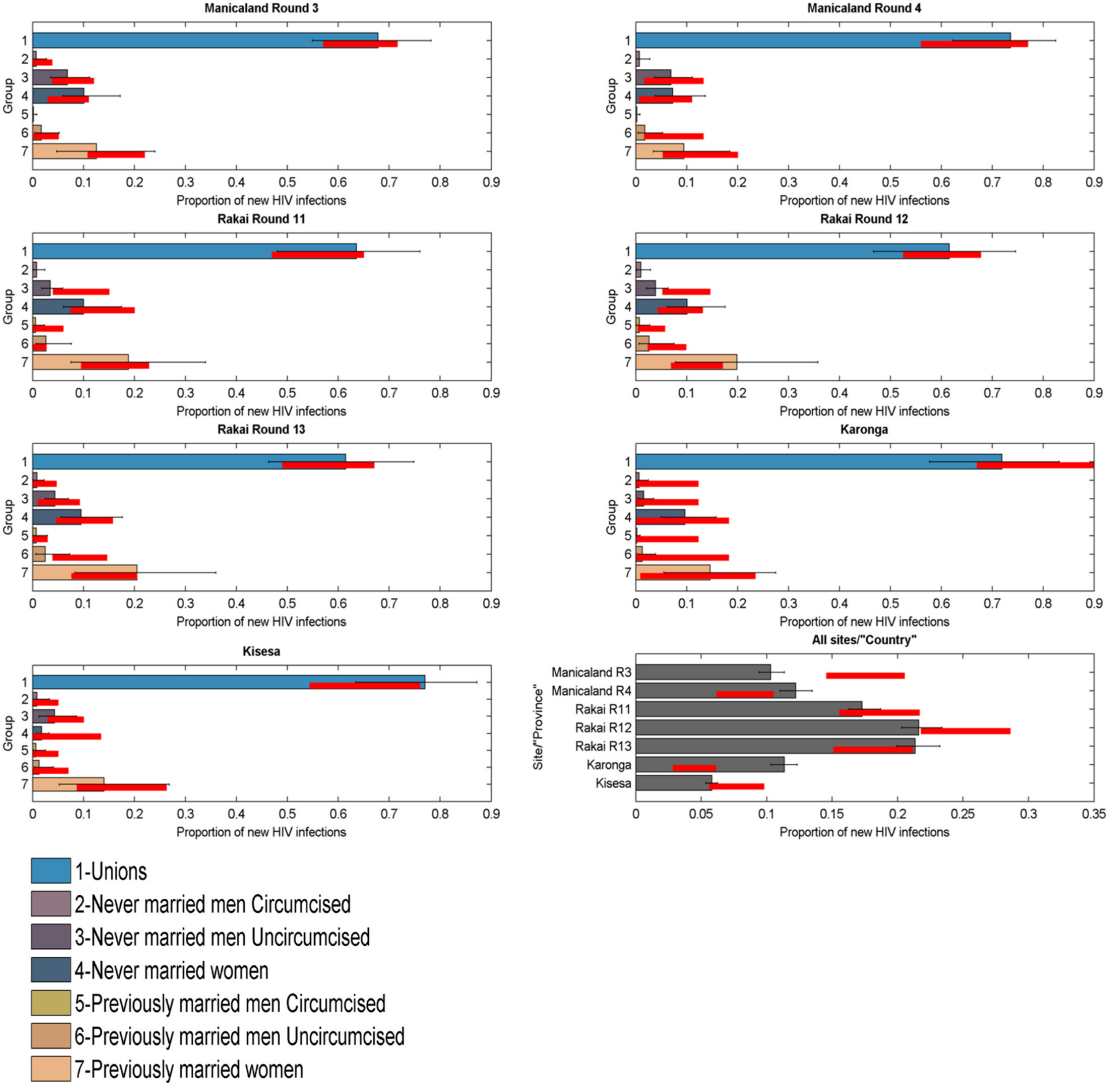
Incidence Patterns Model validation on cohort data from ALPHA Network studies. Distribution of new infections per population group (coloured bars) and round (grey bars), as estimated by the model when modelled as provinces within a country, compared to data (red lines).

To test the model’s ability to predict the distribution of new infections by group, it was applied to each round of data in each of the four ALPHA Network studies. The 95% credible intervals of the model’s projections overlapped with the 95% confidence intervals of the data for all groups, suggesting that the model performs well. Fig 3 presents results obtained when applying the model simultaneously to seven rounds of data from the ALPHA Network studies as if they were provinces within a country. The last panel shows the estimated distribution of new infections by “province” (i.e., round) compared to the data. The model’s 95% credible intervals overlap with the data confidence intervals for four out of seven “provinces”, and the absolute difference between the intervals is under 5% for the other two rounds.

**Fig 4:**
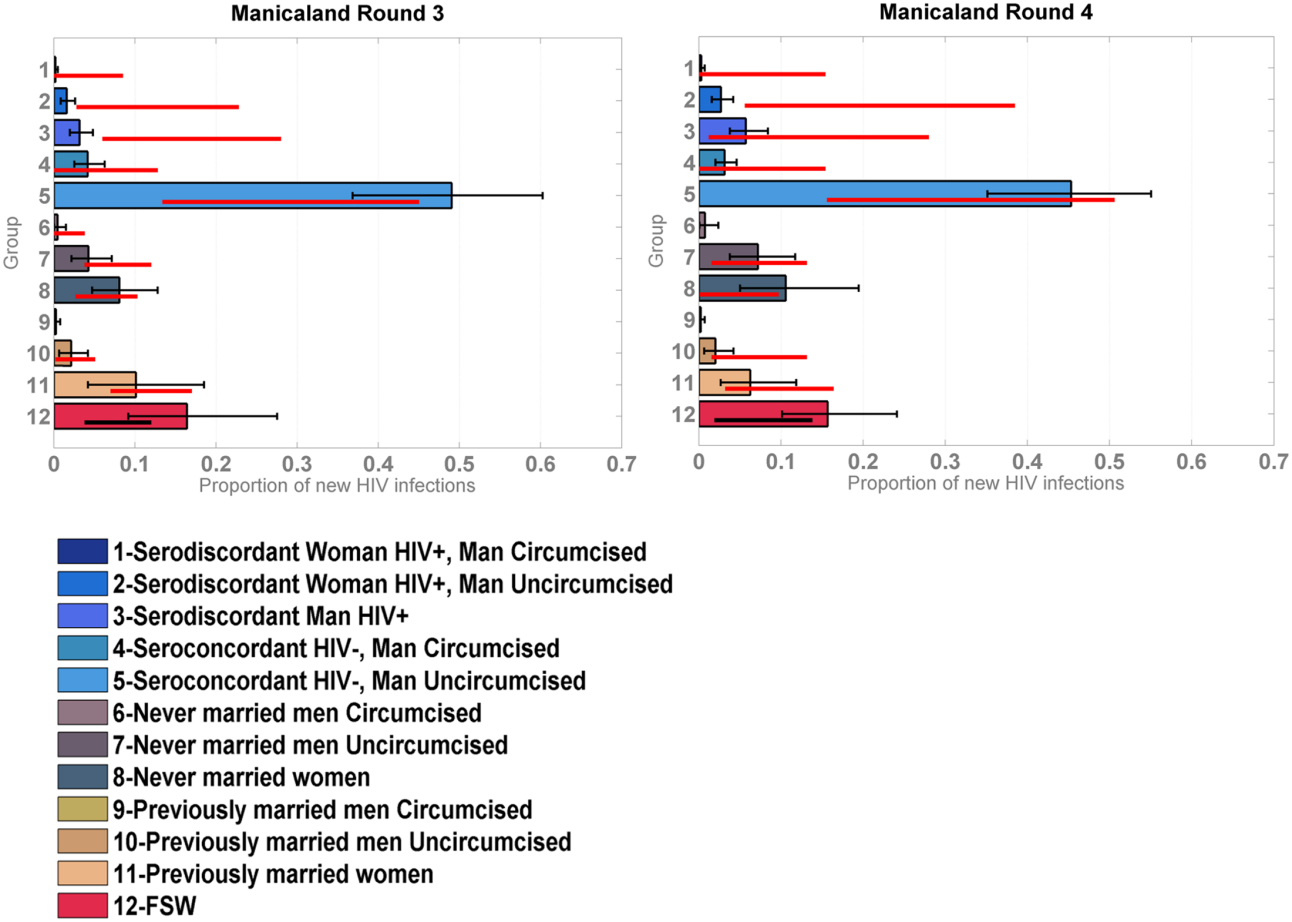
Incidence Patterns Model validation on cohort data from the Manicaland study disaggregated for sex work. Distribution of new infections by group, including FSW, in Manicaland round 3 and 4 estimated by the model (coloured bars) compared to the data (red bars for marital/sexual activity groups and black bars for FSW). FSW, female sex workers.

The model was reapplied to the Manicaland data after stratifying for FSW. As shown in Fig 4, which also provides a more detailed stratification of the unions, the model captured the contribution of FSW to the number of new infections with reasonable accuracy; the confidence intervals of the data overlapped with the credible intervals of the model for both rounds of data.

**Fig 5:**
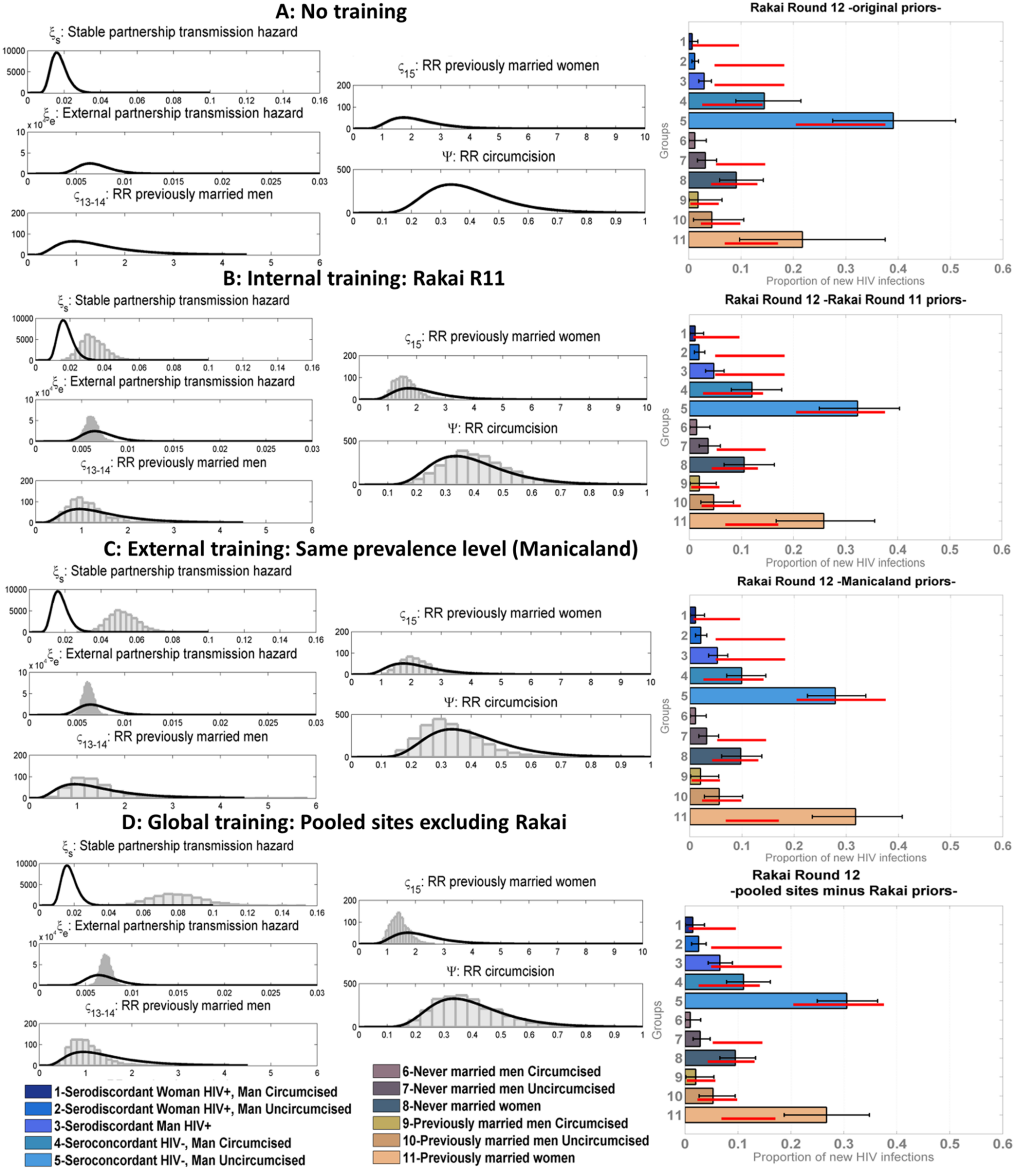
Proportion of new infections estimated by the model compared to data for round 12 of the Rakai cohort using different sets of incidence priors. Mean model estimates and 95% credible intervals are shown in grey, 95% confidence intervals of the data in red. On the left of the figure, the original set of incidence prior distributions (lines) used in the “no training” scenario (A) is compared to the “updated prior distributions” (histograms) used in the different training scenarios: internal training (B), external training (C), and global training (D). The *x*-axes on these graphs correspond to the values for each of the priors, with the stable and external partnership transmission hazards, shown as cases per person-years at risk, and the *y*-axes correspond to the frequency of value intervals, shown as number of iterations. The sets of “updated priors” in (B–D) were obtained from training the model on round 11 of Rakai, rounds 3 and 4 of Manicaland, and all pooled sites excluding Rakai, respectively. RR, relative risk.

Fig 5 shows the results of the model validation for round 12 of Rakai with original priors and various training strategies. The original priors and posteriors obtained after training the model and then used as “updated priors” in these analyses are plotted on the left. Following each training, the within partnership infection hazard was shifted to the right of the original prior distribution, suggesting it is significantly higher than observed in the trials used to derive the original priors. This was expected as trials are highly controlled contexts where the best standards of preventive care are offered to all participants. The range estimated varied from 0.02/person-years at risk to 0.16/person-years at risk depending on the training method. The range of the infection hazard from external partners was within the ranges estimated in trials but narrower. The original and trained priors for the efficacy of circumcision were similar for all training types. The estimated relative risk of infection among previously married men and women compared to that in unions was lower in the models with internal and global training with pooled sites compared to the original prior, but slightly higher when training the model on Manicaland data; however, the distributions overlapped in all cases.

The patterns of distribution of new infections and the performance of the model differed according to the training method. The mean posterior log-likelihood for each type of training is shown in Table 5, for all rounds. The training methods are ranked according to which resulted in the maximum mean posterior log-likelihood; the last column in Table 5 shows the best training method and training methods with results within five log-likelihood units of the best one. Overall, any form of training, and especially training based on all the data from the sites combined, led to significant improvements in model performance. For example, using Rakai data from round 12 (presented in Fig 5 and highlighted in bold in Table 5), the model with global training performed best, followed by the models with internal training, external training, and no training. The difference between the best training method and the other three was more than five log-likelihood units, and thus the global training method was considered to be significantly better. On all rounds of data, the globally trained model performed best or was amongst the best set of training methods. External training performed similarly to global training. In all but one setting (Kisesa), the trained models were significantly better than the original model.

**Table 5:** Mean posterior log-likelihood of the data for each round and site given the model and different sets of priors.

Site and Round	Training Method				Ranking of Training Methods	Within 5 Log-Likelihood Units of Best Method
	1: No Training	2: External Training	3: Global Training	4: Internal Training		
Manicaland round 3	−79.8	−36.3	−32.5	NA	3, 2, 1	3, 2
Manicaland round 4	−43.8	−31.4	−29.8	−35.5	3, 2, 4, 1	3, 2
Rakai round 11	−57.3	−48.1	−42.6	NA	3, 2, 1	3
**Rakai round 12**	**−81.0**	**−64.6**	**−56.8**	**−63.6**	**3, 4, 2, 1**	**3**
Rakai round 13	−37.4	−31.0	−28.6	−27.3	4, 3, 2, 1	4, 3, 2
Kisesa	−12.3	−12.7	−11.8	NA	3, 1, 2	3, 2, 1
Karonga	−16.5	−16.7	−11.4	NA	3, 1, 2	3

For the log-likelihood, a higher value means a better agreement between data and model. Rakai data from round 12 in bold.

NA, not applicable.

### Application to Countries in the Region

#### HIV acquisition

**Fig 6:**
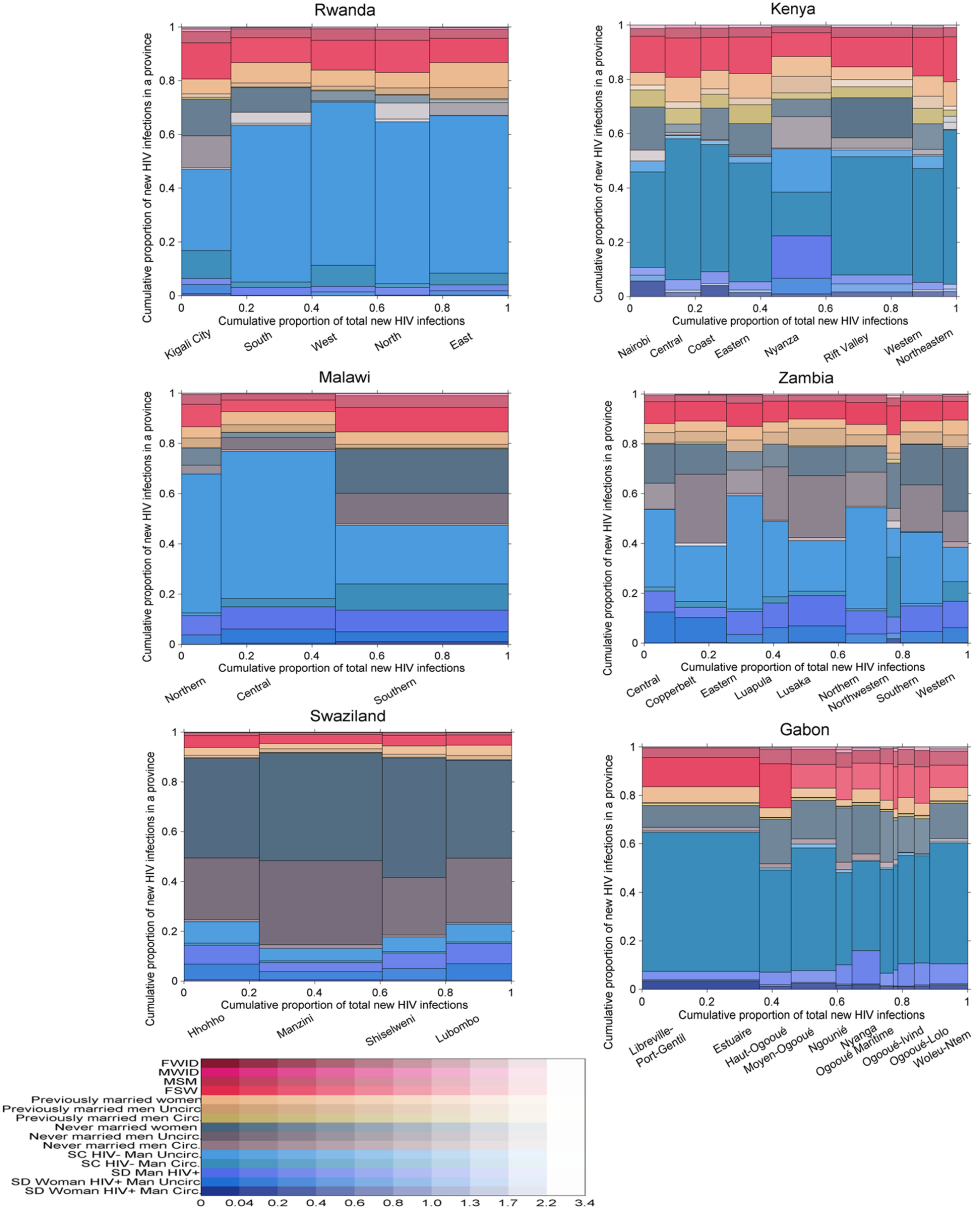
Estimated proportion of new infections acquired by province and group in 6 sub-Saharan African countries. Each graph is divided vertically into provinces, and each province is divided horizontally into population groups shown in different colours. The area of the bars is proportional to the number of new infections, allowing an appreciation of both the relative importance of each province in terms of total incidence and of the contribution of each group within each province. Uncertainty is presented using a transparency gradient corresponding to the posterior coefficient of variation: the higher the transparency, the higher the uncertainty of the particular estimate. circ., circumcised; FSW, female sex workers; FWID, women who inject drugs; MSM, men who have sex with men; MWID, men who inject drugs; SC, sero-concordant; SD, sero-discordant; uncirc., uncircumcised.

The posterior distribution of new infections in Gabon, Kenya, Malawi, Rwanda, Swaziland, and Zambia by province and population group using “updated priors” from the global training on all ALPHA Network studies is shown in Fig 6. The incidence patterns by group were relatively similar between all countries except Swaziland. In the five countries other than Swaziland, individuals in unions contributed to a high share (40% to 75%) of new infections, with a majority of these occurring among sero-concordant HIV-negative couples. Never married women accounted for <5% to >20% of new infections, while the contribution of never married men varied more broadly, from very low in Gabon and Rwanda to high in Zambia, with nearly 30% of new infections occurring in this group in some provinces. Previously married men and women contributed to less than 10% of new infections in all countries except Kenya, where their contribution was slightly higher. FSW accounted for between 5% and nearly 20% of new infections, MSM for up to 5% of new infections, and MWID and FWID for less than 1%.

Swaziland exhibited different incidence patterns. The majority of new infections were estimated to occur among never married men and women, with only 15% to 25% occurring among individuals in unions. This difference can be explained by the demographic and prevalence patterns: fewer men and women reported being in union in Swaziland, and a higher proportion of these were sero-concordant HIV-positive and sero-discordant compared with the other countries (see S4 and S5 Figs for a graphical representation of demographic and HIV prevalence patterns in each country). In addition, HIV prevalence was high among never married men and women, and there was a low prevalence of circumcision among men and a relatively short mean duration of sexual activity among both men and women, giving rise to this pattern. The proportion of new infections occurring in key populations was also lower than in the other countries, reflecting a more generalised epidemic.

Within countries, the distribution of new infections was relatively homogeneous across provinces with a few exceptions: in the southern province of Malawi, individuals in unions had a lower contribution to new infections than in the other two provinces, while never married men and women and FSW had a higher contribution. A similar pattern was observed in Kigali City, Rwanda. Nyanza, in Kenya, also differed from the other provinces, with a higher contribution of never married men, reflecting lower circumcision prevalence and a different distribution of couples by sero-concordance and circumcision status.

The geographical distribution of new infections was heterogeneous, mostly reflecting differences in population sizes. However, some provinces, including Nyanza in Kenya and Manzini in Swaziland, were estimated to contribute a disproportionately high proportion of new infections for their population sizes.

Uncertainty in the results was higher among key populations than among other population groups in all six countries and among never married men in Rwanda, Kenya, Zambia, and Gabon. There was also high uncertainty in the estimates among previously married men in Kenya, reflecting low sample sizes.

#### HIV transmission

**Fig 7:**
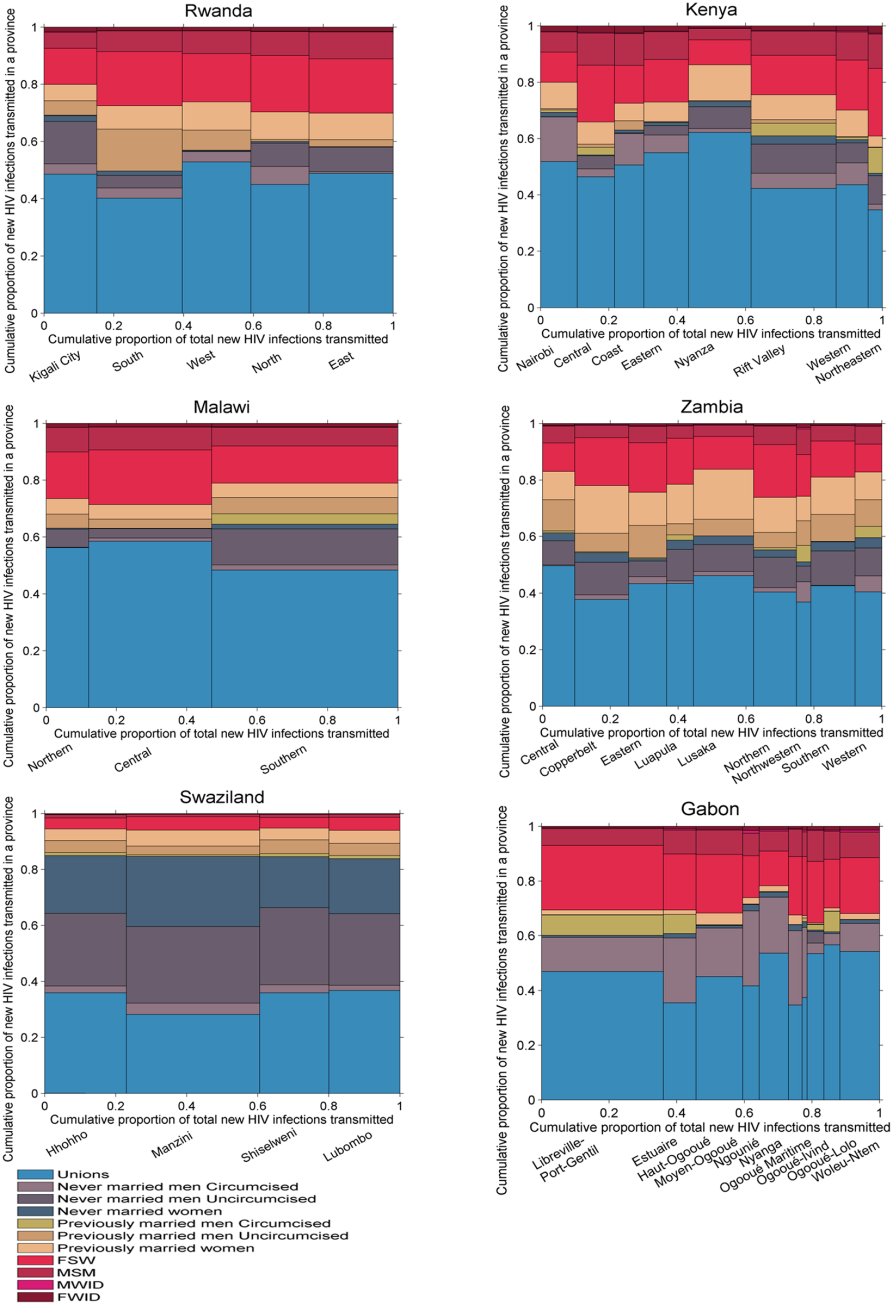
Proportion of new infections transmitted by province and population group in six sub-Saharan African countries. FSW, female sex workers; FWID, women who inject drugs; MSM, men who have sex with men; MWID, men who inject drugs.

Fig 7 shows the distribution of new infections transmitted by each group in each of the countries studied given assumptions on transmissibility and mixing patterns in the population. In Gabon, Kenya, Malawi, Rwanda, and Zambia, previously married men, FSW, and MSM represented only a small fraction of acquired infections but they had a larger contribution to transmission. Conversely, never married women, who represented a relatively large proportion of new infections, contributed much less to onwards transmission. Individuals in unions contributed to a lower or equivalent proportion of transmissions compared to their contribution to the number of infections acquired.

The transmission patterns were different in Swaziland, where individuals in unions contributed to a higher proportion of transmissions than they did to the numbers of new infections acquired, as a result of the high HIV prevalence in unions. Both never married men and never married women accounted for a high proportion of infections transmitted, also as a result of high HIV prevalence in these groups.

## Discussion

### Findings, Interpretation, and Contribution to the Field

Modelling tools to estimate the distribution of new infections in the population are needed to inform HIV prevention, screening, and treatment planning at the local level [8]. We built on existing approaches [42–44] to develop a new version of the MoT model, widely used in HIV policy. Our model, the IPM, relies on available, high-quality data obtained from DHS, IBBS, and local surveys to predict the distribution of new infections according to key determinants of risk: marital/sexual activity status, HIV and ART status within unions, circumcision status, membership in key populations based on risk behaviours, and geographical location. Model validation using cohort data from four settings in the region—Manicaland, Zimbabwe; Karonga, Malawi; Kisesa, Tanzania; and Rakai, Uganda [17]—showed that the model accurately predicts the distribution of new infections acquired in the general population according to key characteristics including geographical location and that its projections can be improved by training it on historical cohort data from other settings in the region.

As such, the IPM offers substantial improvements over the MoT model, both in terms of methodological rigour and programmatic relevance. Its population structure among individuals in unions considers important heterogeneities in risk that determine incidence patterns and therefore limits the risk of overestimating their contribution to the epidemic. The IPM uses reliable and widely available data on HIV prevalence and risk determinants to estimate incidence, therefore minimising the biases and uncertainty associated with self-reported behaviour [45]. Unlike the MoT model, the IPM is implemented within a Bayesian framework that uses prior information reflecting current knowledge of demography and HIV prevalence in SSA, minimising the impact of small datasets on the results, but also allowing for a systematic representation of uncertainty. Finally, a key methodological improvement offered by our model is its validation. Validation is a critical stage in the modelling process, and this study contributes to ongoing efforts to improve the assessment of model performance—through model-to-model comparisons and model-to-data comparisons—by initiatives such as the HIV Modelling Consortium [46–48].

To provide examples of model outputs, we applied the model to six countries in the region and compared the distribution of new infections acquired in each of these. Individuals in unions contributed to a high proportion of new infections in all countries, with a lower contribution in Swaziland, where marriage is less common and HIV prevalence in unions is high, reflecting a hyperendemic epidemic. Sero-concordant HIV-negative unions accounted for a large share of new infections in these countries, indicating that interventions promoting condom use with concurrent partners would be key to reducing incidence. In Malawi, Zambia, Swaziland, and the Nyanza province in Kenya, sero-discordant unions accounted for a larger proportion of new infections among unions, suggesting that interventions such as couples HIV counselling that lead to early ART treatment or pre-exposure prophylaxis (PrEP) use are needed. Community interventions to promote couples HIV counselling have been successfully implemented in Zambia [49], Rwanda [50], and elsewhere in the region, testing a range of strategies to maximise impact [51]. In Zambia, especially in the Copperbelt and Lusaka provinces, and in Swaziland, the contribution of never married men was very high, a finding that is partly explained by the low prevalence of circumcision in these countries [52]. Findings from studies investigating the determinants of uptake of voluntary medical male circumcision in these two countries should inform its scale up [53–56], and strategies such as early infant circumcision [57,58] or circumcision among adolescents through school programmes [59], which are likely to have higher acceptability, should be promoted [60]. Never married women were also disproportionately affected in Swaziland compared to their population size (see S2 Fig), and similarly in Zambia, Gabon, the Southern province in Malawi, and Kigali City and the South province in Rwanda. Previously married men accounted for a large proportion of transmissions in these settings according to the transmission model, suggesting that intergenerational sex could be contributing to the high incidence burden among young women. Interventions that delay the onset of sexual activity, promote gender equality, and improve access to services have been shown to be effective at reducing HIV risk among young women in the region [61]. A comprehensive review by the What Works for Women and Girls initiative provides guidance for their scale up [62]. In all countries, FSW contributed disproportionately to incidence compared to their population size, and their importance to prevention was also highlighted by the transmission model outputs. Improving the working conditions of FSW and engaging them in healthcare [63–65] is paramount for controlling the epidemic in the region in the short and long term [66,67]. Similarly, MSM contributed disproportionately to the proportion of infections acquired and transmitted and should be a priority for national HIV programmes in the region [68–70]. Contextual information on risks and interventions among this population is becoming available, providing valuable guidance for programme design and implementation [71–76].

### Study Limitations

There are three main sources of limitations in our study: the model structure, the incidence estimation methods, and the data used to both apply the model in countries and validate it. In terms of model structure, the model’s parsimony, which conveys transparency to the results generated, also implies several necessary simplifications. The model does not include partners of key populations such as FSW clients or stable female partners of MSM. Our decisions were driven by the need to limit categories to groups for which incidence could be estimated in a straightforward way, for which data were available, and that had programmatic relevance. Implementing interventions directed at stable partners of members of key populations is difficult in practice. However, it is important that the contribution of key populations to the epidemic is not underestimated as a result of the model structure. To account for this, we also presented the distribution of infections transmitted, which provides a complementary perspective on incidence patterns, highlighting priorities for prevention. These results are more illustrative than definitive, as they strongly depend on assumptions about mixing patterns in the population. Country- or province-specific information on sexual networks, as well as phylogenetic data providing insights into transmission patterns, could be used to refine the mixing matrix definition, leading to more robust results.

Among the groups that are represented in the model, determinants of HIV risk such as age, age gaps in partnerships [77,78], number of previous marriages, engagement in transactional sex [79,80], exposure to sexual violence [78,81–83], sexual identity for MSM [84,85], and work place for FSW [85,86] are not included. Increased resolution by (social) determinants is hindered by the lack of power once the data are disaggregated further. However, the model results should be combined with evidence from other epidemiological and social sciences studies that identify determinants of infection at the local level.

Although our model represents concurrency, it does not incorporate the role of acute infection [87,88], potentially leading to underestimation of the risk of infection among unions. This is especially important among polygamous unions, which are not explicitly represented in the model. While the risk might be concentrated in some couples, the model applies an average risk from concurrent partnerships among all HIV-negative individuals in unions, failing to represent this heterogeneity. However, the validation shows that the model is able to estimate the distribution of new infections among unions with reasonable accuracy.

In terms of incidence estimation, methods that use prevalence and duration of exposure assume that incidence is constant through time [89], and the model will therefore not detect sudden changes in incidence caused by rapid changes in behaviour or successful interventions. However, these changes are unlikely to occur on a large scale or to be of a high magnitude over a 1-y time scale and so are unlikely to affect the results. The short time scale of the model, however, implies that longer-term epidemic dynamics are not captured [14,67,90]. While this represents a limitation of the model, making long-term projections requires additional data and carries greater uncertainty [46] as sexual behaviours and access to treatment might change in the future. Two essential features of this model are accuracy and applicability on a cross-section of countries, and therefore a long-term perspective would not be appropriate.

Although the data used in this model mostly come from the DHS surveys, which follow a strong methodology, they are still subject to reporting bias, and therefore the results might be affected by this bias. In addition, as in all analyses, the extent to which variances have been constrained by our choices of parametric distribution can understate the overall uncertainty in the model outputs.

The ALPHA Network cohort studies are a valuable source of information for model validation as they benefit from years of experience working with household participants in the context of HIV testing [17]. Nonetheless, their findings are susceptible to reporting bias and inaccuracies in the estimation of sero-conversion dates. After restricting analyses to participants who were present at two subsequent surveys and tested for HIV, the number of sero-conversions was relatively small in some groups, especially among linked unions, leading to broad confidence intervals.

### Implications and Next Steps

Our model is currently tailored to the generalised epidemics of SSA, where the vast majority of HIV cases are observed among the general population. The application of the model by national teams has been piloted in Botswana, and model documentation is being developed based on the feedback received. The model is being implemented on a user-friendly software platform to facilitate its scale up in the region. However, for a number of countries in SSA (17 out of 42 countries), recent DHS data are currently unavailable, preventing them from applying the model (S12 Table). To predict the distribution of new infections in epidemics that are concentrated among key populations, a different type of model would be required. Such a tool should have a higher resolution of the dynamics of transmission within and between key populations and the general population and therefore would require different data.

Within the SSA region, epidemic drivers also vary, and further model testing in other settings is needed to confirm the model’s ability to capture different epidemic dynamics. Importantly, the capacity of the model to accurately estimate the contribution of MSM and PWID to the distribution of new infections needs to be assessed. Data availability was the limiting factor in this analysis, but progress in data collection methods among key populations as well as increased research capacity on MSM in SSA [91–96] should help address this gap in the near future. Model validation is an iterative process that should be carried out as long as the model is in use to improve the quality of its outputs as data become available.

This study provides a highly pragmatic and reliable perspective on incidence patterns highlighting priorities for prevention across geographic areas and populations, an approach that has been repeatedly emphasised as the cornerstone of renewed efforts to drive down infection rates. Modelling tools to inform HIV programme planning that provide for geographical heterogeneity are becoming available: Anderson et al.’s model [43] and Optima[97] are two key examples. They both have a long-term perspective and focus on allocative efficiency. The IPM, in contrast, outputs short-term predictions, which are also needed to guide not only prevention but also testing and treatment programmes, especially in the context of the UNAIDS 90-90-90 target. The IPM was designed to provide clear information on incidence as opposed to guidance on interventions’ cost-effectiveness, and its strength lies in its simplicity, systematic use of information, and careful representation of the population in terms of programmatic relevance. Its outputs should not be directly (i.e., proportionally) translated into budget allocation, as some populations will require more costly interventions, and specific provinces might have low incidence precisely because their HIV programmes are well designed and implemented, requiring sustained funding. Indeed, the model outputs should be contrasted with local programme data obtained through monitoring and evaluation efforts to identify discrepancies between incidence, programme activities, and spending. Contextual epidemiological and cost-effectiveness studies should then be used to guide programmatic planning among the populations predicted to contribute importantly to incidence. The IPM also feeds into the monitoring process by informing data collection priorities as it highlights settings and populations in which uncertainty is high.

To our knowledge, this is the first model to embed prior epidemiological and demographic information from SSA and to be validated against data from a range of settings in the region. We have demonstrated that the model can reliably predict HIV incidence patterns, and that this is enhanced when local high-quality data are incorporated into the analyses. We believe this model is a valuable tool to inform programme planning and that its application would contribute to the formulation of the effective and efficient prevention and testing programmes that are urgently needed to curb the epidemic.

## Supporting Information

S1 Fig Model diagram describing the stratification of the population in the model by marital/sexual activity status, key population, HIV and ART status within unions, circumcision status, group definitions, and HIV transmission routes considered. The model represents the distribution of the population in each of the provinces by sex, marital/sexual activity status, and circumcision status and requires information on HIV prevalence in each group. Unions are divided by sero-concordance and ART status. The other marital/sexual activity groups represented are never married men and women, previously married men and women (including widowed, divorced, and separated), and men and women not sexually active in the past 12 mo (assumed to be at no risk of infection), and the key populations included are FSW, MSM, and PWID.
(TIF)

S2 Fig Trace plots for incidence parameters for the validation of the model on all ALPHA Network sites. (PDF)

S3 Fig Trace plots for parameters determining the ART coverage, duration of sexual activity, distribution of the total population by population group, and HIV prevalence by population group. (PDF)

S4 Fig Estimated distribution of the total population by population group and province in six sub-Saharan African countries. Each graph is divided vertically into provinces, and each province is divided horizontally into population groups shown in different colours.
(TIF)

S5 Fig Estimated distribution of HIV prevalence by population group and province in six sub-Saharan African countries. Each graph is divided vertically into provinces, and each province is divided horizontally into population groups shown in different colours.
(TIF)

S1 Table Distribution of the population among couples, men, and women according to risk determinants. (PDF)

S2 Table Countries included in the DHS meta-analysis to obtain prior values for each sub-region of sub-Saharan Africa. (PDF)

S3 Table Distribution of new infections by gender, marital/sexual activity status, and union type in round 3 of the Manicaland cohort. (PDF)

S4 Table Distribution of new infections by gender, marital/sexual activity status, and union type in round 4 of the Manicaland cohort. (PDF)

S5 Table Distribution of new infections by gender, marital/sexual activity status, and union type in round 11 of the Rakai cohort. (PDF)

S6 Table Distribution of new infections by gender, marital/sexual activity status, and union type in round 12 of the Rakai cohort. (PDF)

S7 Table Distribution of new infections by gender, marital/sexual activity status, and union type in round 13 of the Rakai cohort. (PDF)

S8 Table Distribution of new infections by gender, marital/sexual activity status, and union type in round 1 of the Karonga cohort. (PDF)

S9 Table Distribution of new infections by gender, marital/sexual activity status, and union type in round 5 of the Kisesa cohort. (PDF)

S10 Table Data for Gabon, Kenya, Malawi, Rwanda, Swaziland, and Zambia by province. (PDF)

S11 Table Sample sizes of the data for Gabon, Kenya, Malawi, Rwanda, Swaziland, and Zambia by province. (PDF)

S12 Table Latest Demographic Health Surveys implemented in sub-Saharan African countries. (PDF)

S13 Table Priors for the parameters informing the transmission model. (PDF)

S1 Text Supplementary information. (PDF)

## Abbreviations

ART: antiretroviral therapy
DHS: Demographic and Health Surveys
FSW: female sex workers
FWID: women who inject drugs
IBBS: Integrated Behavioural and Biological Surveys
IPM: Incidence Patterns Model
MCMC: Markov chain Monte Carlo
MoT: Modes of Transmission
MSM: men who have sex with men
MWID: men who inject drugs
PWID: people who inject drugs
SSA: sub-Saharan Africa
STI: sexually transmitted infection
