# Supplementary figures and images for: The Incidence Patterns Model to Estimate the Distribution of New HIV Infections in Sub-Saharan Africa: Development and Validation of a Mathematical Model
Source: PLoS Med. 2016 Sep 13;13(9):e1002121. doi: 10.1371/journal.pmed.1002121 (PMC5021265; doi:10.1371/journal.pmed.1002121)

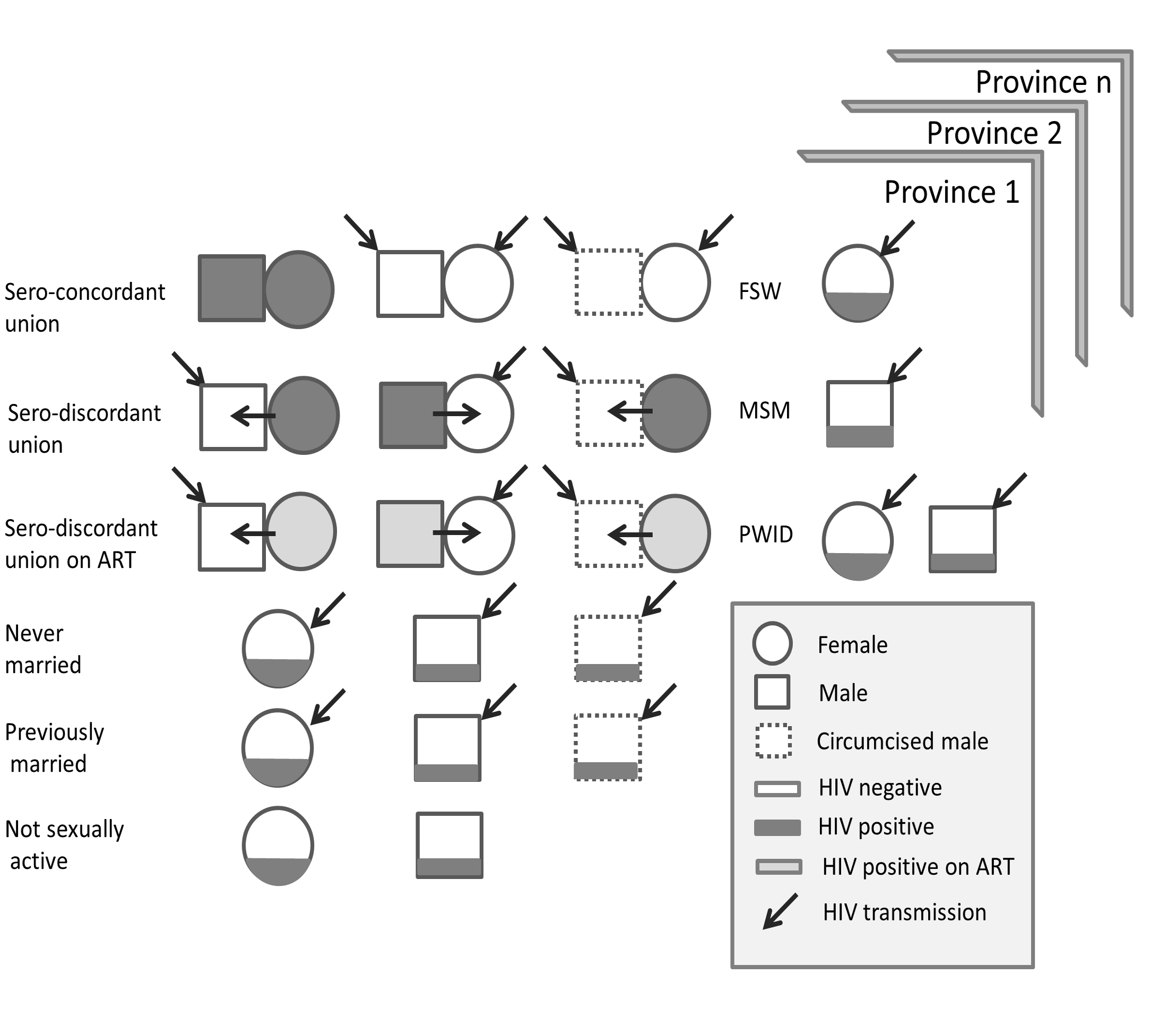

Supplement: S1 Fig — The model represents the distribution of the population in each of the provinces by sex, marital/sexual activity status, and circumcision status and requires information on HIV prevalence in each group. Unions are divided by sero-concordance and ART status. The other marital/sexual activity groups represented are never married men and women, previously married men and women (including widowed, divorced, and separated), and men and women not sexually active in the past 12 mo (assumed to be at no risk of infection), and the key populations included are FSW, MSM, and PWID. (TIF) [file pmed.1002121.s001.tif]

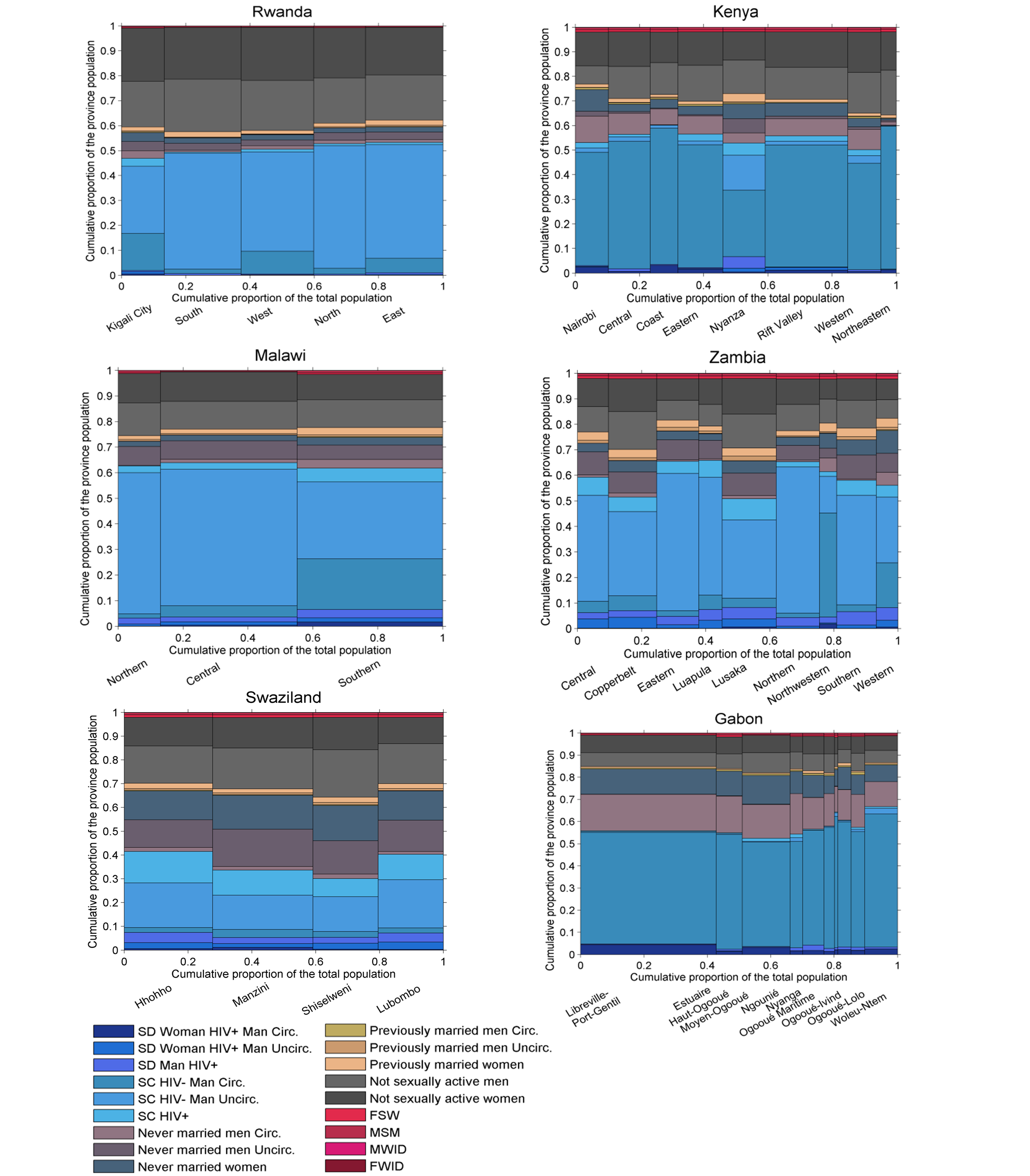

Supplement: S4 Fig — Each graph is divided vertically into provinces, and each province is divided horizontally into population groups shown in different colours. (TIF) [file pmed.1002121.s004.tif]

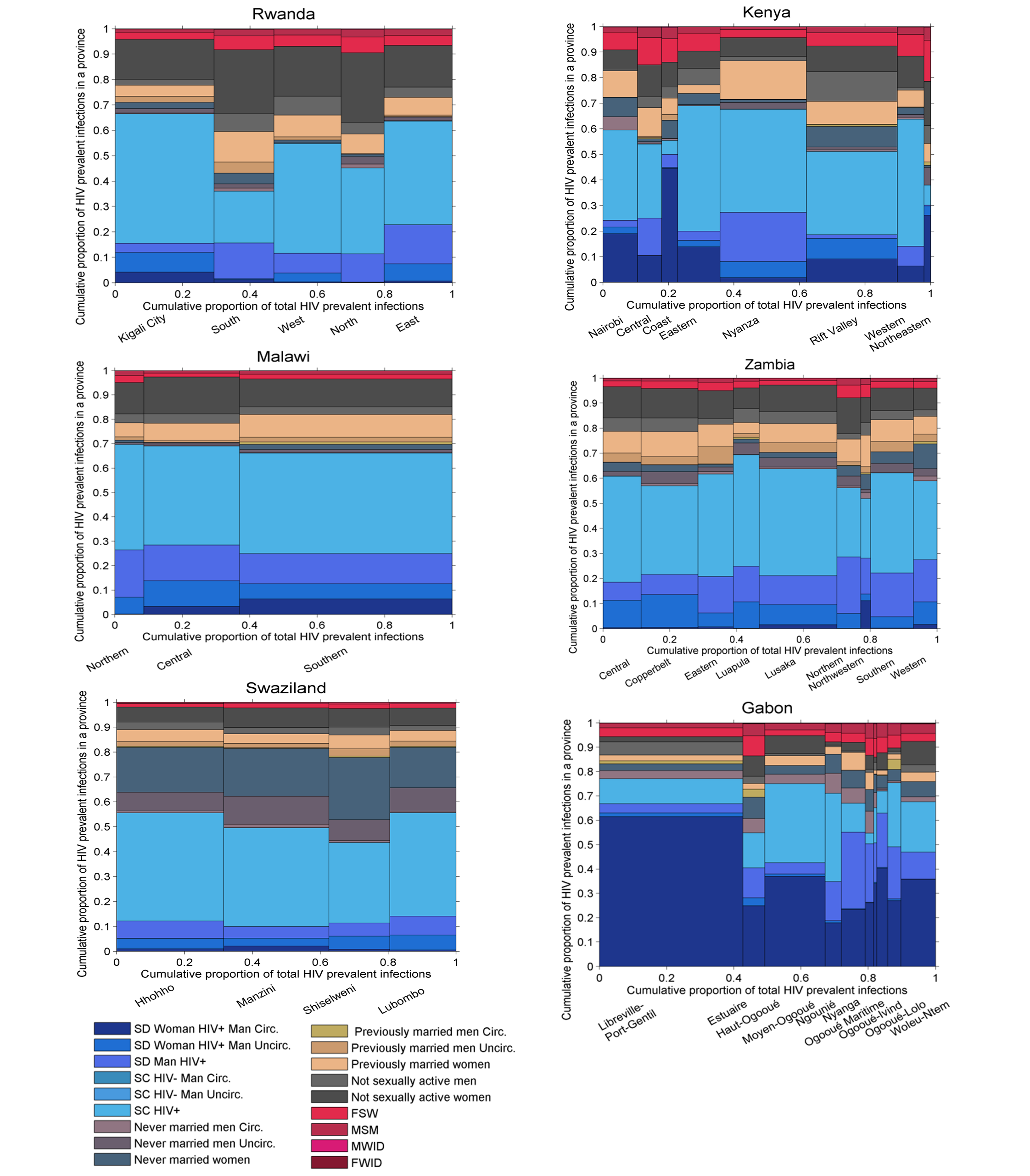

Supplement: S5 Fig — Each graph is divided vertically into provinces, and each province is divided horizontally into population groups shown in different colours. (TIF) [file pmed.1002121.s005.tif]
