# Supplementary material for: The Incidence Patterns Model to Estimate the Distribution of New HIV Infections in Sub-Saharan Africa: Development and Validation of a Mathematical Model
Source: PLoS Med. 2016 Sep 13;13(9):e1002121. doi: 10.1371/journal.pmed.1002121 (PMC5021265; doi:10.1371/journal.pmed.1002121)
Supplement: S2 Fig — (PDF) [file pmed.1002121.s002.pdf]

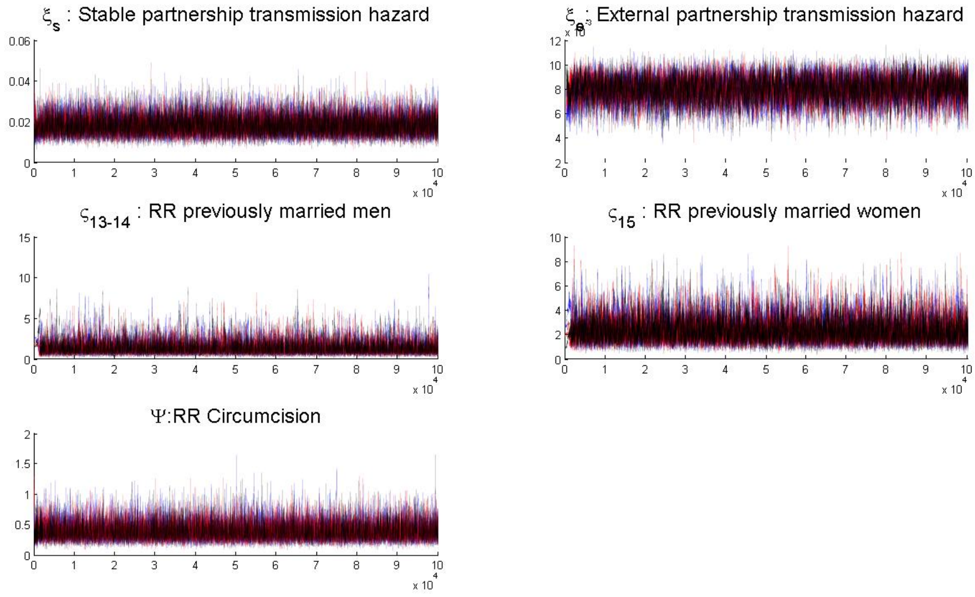


**S2 Fig. Trace plots for incidence parameters for the validation of the model on all Alpha network sites.**
