# Supplementary material for: The Incidence Patterns Model to Estimate the Distribution of New HIV Infections in Sub-Saharan Africa: Development and Validation of a Mathematical Model
Source: PLoS Med. 2016 Sep 13;13(9):e1002121. doi: 10.1371/journal.pmed.1002121 (PMC5021265; doi:10.1371/journal.pmed.1002121)
Supplement: S3 Fig — (PDF) [file pmed.1002121.s003.pdf]

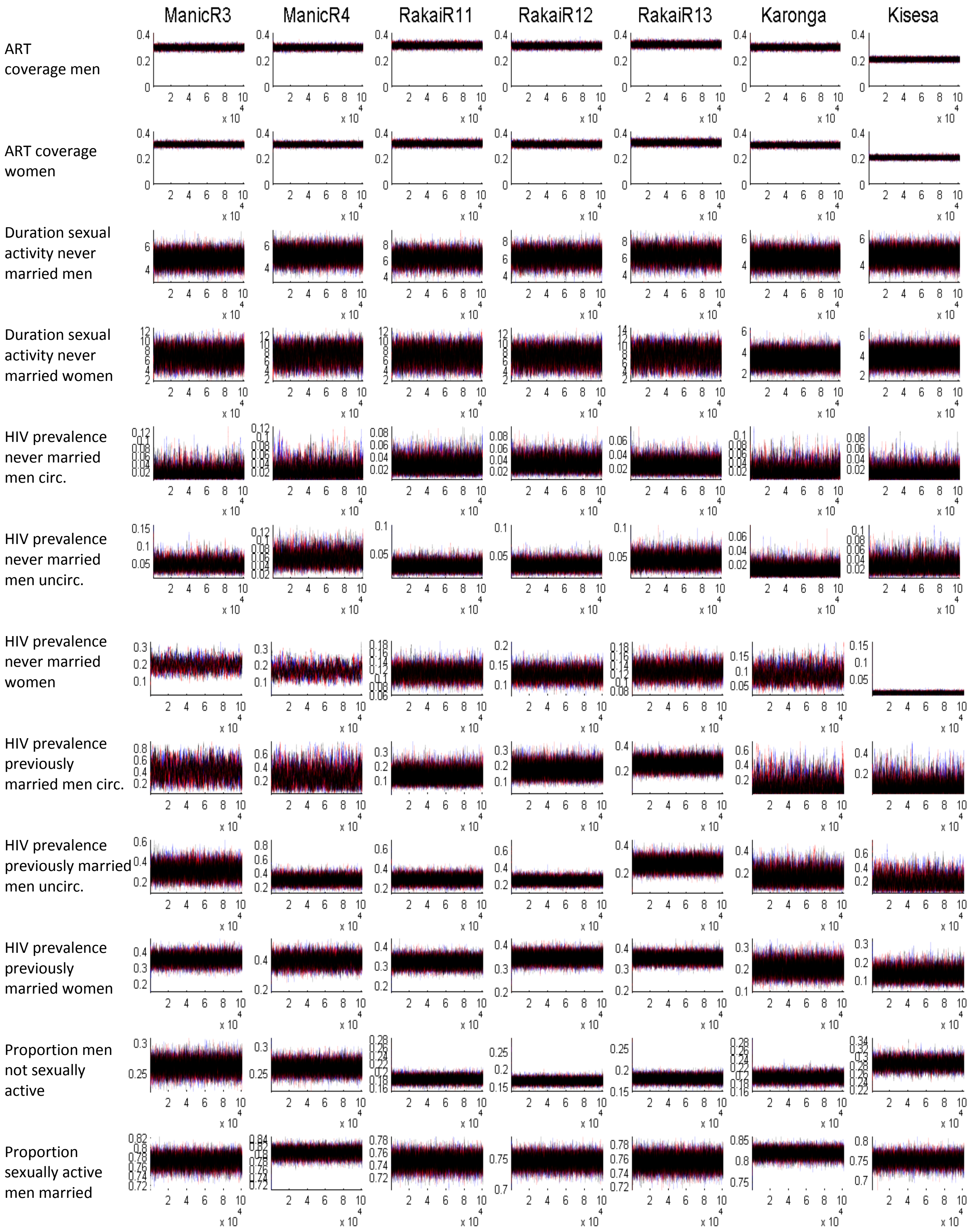

S3 Fig. Trace plots for parameters determining the ART coverage, duration of sexual activity, distribution and prevalence per group.

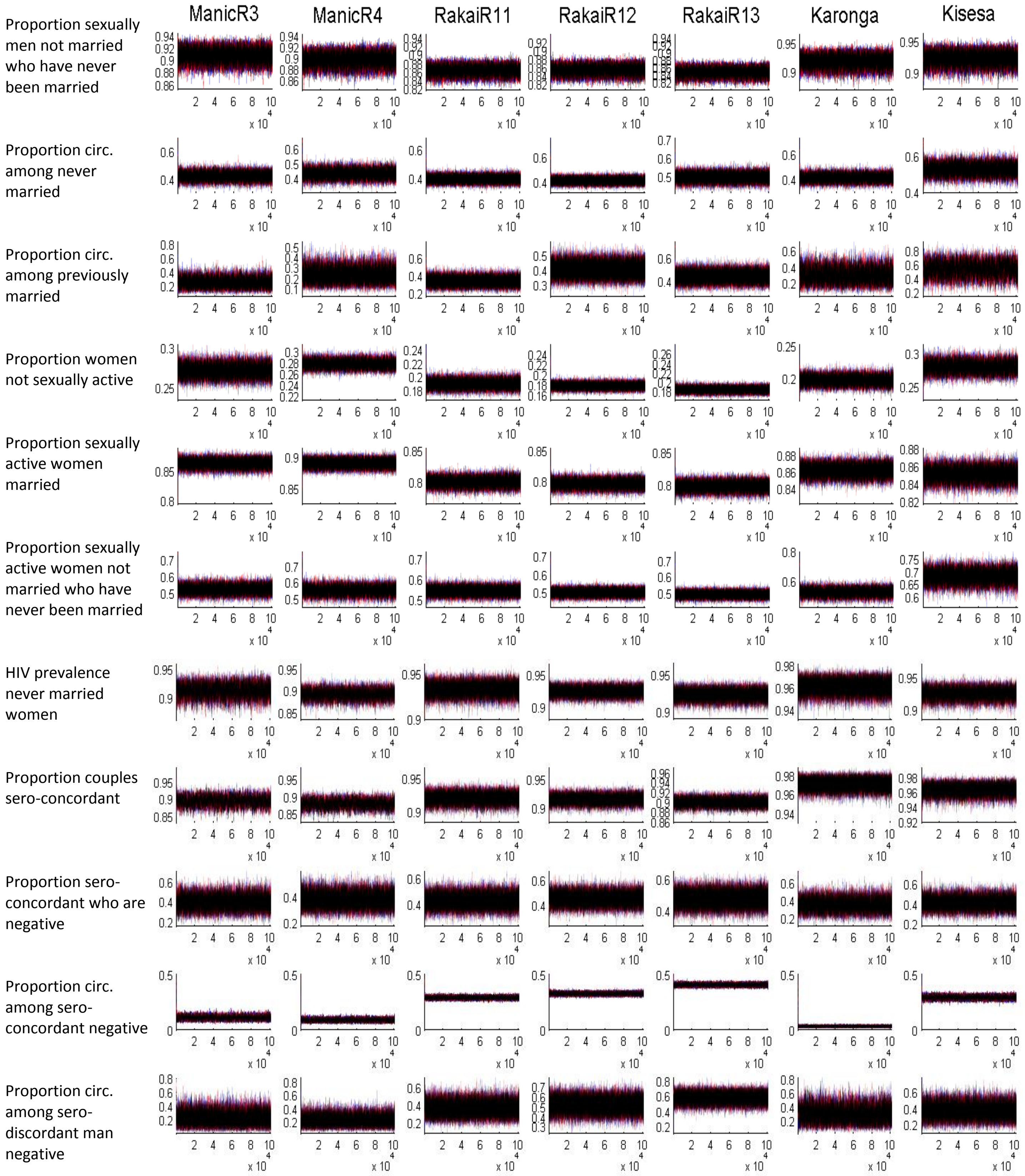

S3 Fig ctd. Trace plots for parameters determining the ART coverage, duration of sexual activity, distribution and prevalence per group.
