# Supplementary material for: The Incidence Patterns Model to Estimate the Distribution of New HIV Infections in Sub-Saharan Africa: Development and Validation of a Mathematical Model
Source: PLoS Med. 2016 Sep 13;13(9):e1002121. doi: 10.1371/journal.pmed.1002121 (PMC5021265; doi:10.1371/journal.pmed.1002121)
Supplement: S1 Table — (PDF) [file pmed.1002121.s006.pdf]

Unions (C)	Risk determinants
1	Sero-discordant, man negative, circumcised
2	Sero-discordant, man negative, uncircumcised
3	Sero-discordant, man positive
4	Sero-concordant negative, man circumcised
5	Sero-concordant negative, man uncircumcised
6	Sero-concordant positive
Men (M)	
1	Married/cohabiting
2	Never married circumcised
3	Never married uncircumcised
4	Previously married circumcised
5	Previously married uncircumcised
6	Not sexually active in past 12 months
Women (W)	
1	Married/cohabiting
2	Never married
3	Previously married
4	Not sexually active in past 12 months

S1 Table. Distribution among couples, men and women according to risk determinants

Symbol	Parameter definition	Rwanda	Gabon	Zambia	Kenya	Malawi	Swaziland	Distribution	Sample Size	Reference
**Mixing Matrix**										
χ_{38}	MSM	0.1	0.15	0.15	0.1	0.1	0.1	Beta	100	Based on the fact that mixing among MSM is very assortative
χ_{39}	MSM that are with married women	0.3	0.3	0.3	0.3	0.3	0.3	Beta	100	Based on proportion of MSM who report being married
χ_{40}	MSM that are not with married women that are with never married women	0.3	0.3	0.3	0.2	0.2	0.3	Beta	100	Assumption
χ_{41}	MSM that are not with married women that are not with never married women that are with previously married women	0.2	0.2	0.2	0.2	0.2	0.2	Beta	100	Assumption
χ_{42}	MSM that are not with married women that are not with never married women that are not with previously married women that are with MWID	0.02	0.02	0.02	0.02	0.02	0.02	Beta	100	Assumption
χ_{43}	MSM that are not with married women that are not with never married women that are not with previously married women that are not with MWID that are with FSW	0.9	0.9	0.9	0.8	0.8	0.8	Beta	100	Assumption
χ_{44}	FSW that are not with never married men	0.1	0.1	0.1	0.1	0.1	0.1	Beta	100	Based on the fact that the majority of men reporting paying for sex are married men
χ_{45}	FSW that are not with never married men that are with married men	0.9	0.8	0.9	0.9	0.9	0.8	Beta	100	Assumption
χ_{46}	FSW that are not with never married men that are not with married men that are with previously married men	0.95	0.95	0.95	0.95	0.95	0.95	Beta	100	Assumption
χ_{47}	FSW that are not with never married men that are not with married men that are not with previously married men that are with MWID	0.2	0.2	0.2	0.2	0.2	0.2	Beta	100	Assumption
	**Transmissibility degree among:**							**Distribution**	**Standard deviation**	
$T_{1\text{-}4}$	Serodiscordant positive unions where the woman is positive	0.1	0.1	0.1	0.1	0.1	0.1	Normal	0.02	Baseline
$T_{5\text{-}8}$	serodiscordant positive unions where the man is positive	0.2	0.2	0.2	0.2	0.2	0.2	Normal	0.04	2 folds higher transmission probability compared to women
T_{9}	Seroconconrdant positive unions	0.15	0.15	0.15	0.15	0.15	0.15	Normal	0.03	Average between transmissibility in serodiscordant unions where man and woman is positive respectively
$T_{10\text{-}11}$	Never married men	0.16	0.16	0.16	0.16	0.16	0.16	Normal	0.032	Higher condom use than among married men and two folds transmission probability compared to women
T_{12}	Never married women	0.08	0.08	0.08	0.08	0.08	0.08	Normal	0.016	Higher condom use than among married women
$T_{13\text{-}14}$	Previously married men	0.2	0.2	0.2	0.2	0.2	0.2	Normal	0.04	Assumed to be equal to that among married men
T_{15}	Previously married women	0.1	0.1	0.1	0.1	0.1	0.1	Normal	0.02	Assumed to be equal to that among married women
T_{18}	FSW	0.1	0.1	0.1	0.1	0.1	0.1	Normal	0.02	Assumed to be equal to that among married women (fewer sex acts/higher condom use)
T_{19}	FWID	0.2	0.2	0.2	0.2	0.2	0.2	Normal	0.04	Assumed to be higher than among married women
T_{20}	MSM	0.3	0.3	0.3	0.3	0.3	0.3	Normal	0.06	Assumed to be higher than among married men (anal sex)
T_{21}	MWID	0.2	0.2	0.2	0.2	0.2	0.2	Normal	0.04	Assumed to be equal to that among married men

Table S13 ctd. Priors for the parameters informing the transmission model

References

1. Wringe A, Floyd S, Kazooba P, et al. Antiretroviral therapy uptake and coverage in four HIV community cohort studies in sub-Saharan Africa. *Tropical Medicine & International Health* 2012; **17**(8): e38-e48.
