# Supplementary material for: The Incidence Patterns Model to Estimate the Distribution of New HIV Infections in Sub-Saharan Africa: Development and Validation of a Mathematical Model
Source: PLoS Med. 2016 Sep 13;13(9):e1002121. doi: 10.1371/journal.pmed.1002121 (PMC5021265; doi:10.1371/journal.pmed.1002121)
Supplement: S2 Table — (PDF) [file pmed.1002121.s007.pdf]

Sub-region	Countries
Western SSA	Gabon
	Cote D’Ivoire
	Mali
	Niger
	Sierra Leone
	Guinea
	Senegal
	Cameron
	Sao Tome
	Congo
Central SSA	Zambia
	Malawi
	Zimbabwe
Eastern SSA	Kenya
	Burundi
	Rwanda
	Ethiopia
Southern SSA	Swaziland
	Lesoto

S2 Table. Countries included in the DHS meta-analysis to obtain prior values for each sub-region of sub-Saharan Africa (SSA)
