# Supplementary material for: The Incidence Patterns Model to Estimate the Distribution of New HIV Infections in Sub-Saharan Africa: Development and Validation of a Mathematical Model
Source: PLoS Med. 2016 Sep 13;13(9):e1002121. doi: 10.1371/journal.pmed.1002121 (PMC5021265; doi:10.1371/journal.pmed.1002121)
Supplement: S3 Table — (PDF) [file pmed.1002121.s008.pdf]

Manicaland R3	Sample size	Percent	Proportion HIV+	Mean duration sexual	Seroconversions (SC)	Rescaled SC	ART coverage HIV+
**Men**							
Not Sexually active	503	27.71	0.06		0	0.0	
Married	1040	57.30	0.18		38	54.7	
Never married circ	14	0.77	0.00	4.2	2	2.9	
Never married uncirc.	209	11.52	0.04		9	12.9	
Previously married circ.	3	0.17	1.00		0	0.0	
Previously married uncirc.	46	2.53	0.28		3	4.3	
Total	1815	100			52	74.8	16.3% (n=240)
**Women**							
Not Sexually active	999	29.31	0.22		0	0.0	
Married	2139	62.76	0.14		66	50.6	
Never married	59	1.73	0.27	8.7	15	11.5	
Previously married	211	6.19	0.35		35	26.8	
Total	3408	100			116	88.9	26.9% (n=605)
**Unions**							
SC Pos.	45	8.51	1		0	0.0	
SC Neg. Man circ	42	7.92	0		1	4.4	
SC Neg. Man uncirc	388	73.36	0		13	57.0	
SD Man pos.	35	6.62	0.5		6 (F)	26.3	
SD Woman pos. Man circ	0	0.00	0.5		0	0.0	
SD Woman pos. Man	19	3.59	0.5		4 (M)	17.5	
Total	529	100.00			24	105.2	

SC: sero-concordant; SD:sero-discordant; pos: HIV positive; circ: circumcised; uncirc: uncircumcised
