# Supplementary material for: The Incidence Patterns Model to Estimate the Distribution of New HIV Infections in Sub-Saharan Africa: Development and Validation of a Mathematical Model
Source: PLoS Med. 2016 Sep 13;13(9):e1002121. doi: 10.1371/journal.pmed.1002121 (PMC5021265; doi:10.1371/journal.pmed.1002121)
Supplement: S4 Table — (PDF) [file pmed.1002121.s009.pdf]

Manicaland R4	Sample size	Percent	Proportion HIV+	Mean duration sexual activity (variance)	Sero-conversions (SC)	Rescaled SC	ART coverage HIV+
**Men**							
Not Sexually active	641	28.15	0.05		0	0.0	
Married	1376	60.43	0.18		18	26.2	
Never married circ	7	0.31	0.00	4.5	0	0.0	
Never married uncirc.	191	8.39	0.05		4	5.8	
Previously married circ.	4	0.18	0.50		0	0.0	
Previously married uncirc.	58	2.55	0.28		4	5.8	
Total	2277	100			26	37.8	16.3% (n=240)
**Women**							
Not Sexually active	1216	28.67	0.26		0	0.0	
Married	2843	67.04	0.14		34	25.9	
Never married	29	0.69	0.24	9.9	6	4.6	
Previously married	153	3.61	0.39		13	9.9	
Total	4340	100			53	40.4	26.9% (n=605)
**Unions**							
SC Pos.	69	8.85	1		0	0.0	
SC Neg. Man circ.	50	6.41	0		0	0.0	
SC Neg. Man uncirc.	565	72.44	0		7 (4M/3F)	28.0	
SD Man pos.	63	8.08	0.5		2 (F)	8.0	
SD Woman pos. Man circ.	3	0.38	0.5		0	0.0	
SD Woman pos. Man uncirc.	30	3.85	0.5		4(M)	16.0	
Total	780	100			13	52.1	

SC: sero-concordant; SD:sero-discordant; pos: HIV positive; circ: circumcised; uncirc: uncircumcised
