# Supplementary material for: The Incidence Patterns Model to Estimate the Distribution of New HIV Infections in Sub-Saharan Africa: Development and Validation of a Mathematical Model
Source: PLoS Med. 2016 Sep 13;13(9):e1002121. doi: 10.1371/journal.pmed.1002121 (PMC5021265; doi:10.1371/journal.pmed.1002121)
Supplement: S5 Table — (PDF) [file pmed.1002121.s010.pdf]

Rakai R11	Sample size	Percent	Proportion HIV +	mean duration sexual activity (variance)	Sero-conversions (SC)	Rescaled SC	ART coverage HIV+
**Men**							
Not sexually active	535	15%	0.04		0	0.0	
Married	2,257	63%	0.11		24	27.1	13% (n=70)
Never married circ.	156	4%	0.03		0	0.0	
Never married uncirc.	456	13%	0.02	7.0 (25)	10	11.3	
Previously married circ.	58	2%	0.12		3	3.4	
Previously married uncirc.	133	4%	0.26		1	1.1	
Total	3595	100%			38	42.9	
**Women**							
Not sexually active	609	15%	0.21		0	0.0	
Married	3,136	66%	0.09		43	38.6	7.8% (n=52)
Never married	414	9%	0.10	9.1 (50)	18	16.1	
Previously married	372	10%	0.30		21	18.8	
Total	4531	100%				73.5	
**Unions**							
SC pos.	114	6%	1.0		0	0.0	
SC neg. Man circ.	484	25%	0.0		2	3.5	
SC neg. Man uncirc.	1190	62%	0.0		22	38.0	
SD Man pos.	70	4%	0.5		9	15.6	
SD Female pos. Man circ.	16	1%	0.5		1	1.7	
SD Female pos. Man uncirc.	31	2%	0.5		4	6.9	
Total	1905	100%			38	65.7	

SC: sero-concordant; SD:sero-discordant; pos: HIV positive; circ: circumcised; uncirc: uncircumcised
