# Supplementary material for: The Incidence Patterns Model to Estimate the Distribution of New HIV Infections in Sub-Saharan Africa: Development and Validation of a Mathematical Model
Source: PLoS Med. 2016 Sep 13;13(9):e1002121. doi: 10.1371/journal.pmed.1002121 (PMC5021265; doi:10.1371/journal.pmed.1002121)
Supplement: S6 Table — (PDF) [file pmed.1002121.s011.pdf]

| Rakai R12 | Sample size | Percent | Proportion HIV + | mean duration sexual activity (variance) | Sero-conversions (SC) | Rescaled SC | ART coverage HIV+ (n=128) |
|---|---|---|---|---|---|---|---|
| **Men** | | | | | | | |
| Not sexually active | 554 | 13% | 0.03 | | 0 | 0.0 | |
| Married | 2,611 | 64% | 0.10 | | 41 | 49.3 | 16.5% |
| Never married circ. | 214 | 5% | 0.03 | 6.7 (26) | 0 | 0.0 | |
| Never married uncirc. | 498 | 12% | 0.02 | | 13 | 15.6 | |
| Previously married circ. | 88 | 2% | 0.16 | | 4 | 4.8 | |
| Previously married uncirc. | 145 | 4% | 0.22 | | 8 | 9.6 | |
| Total | 4110 | 100% | | | 66 | 79.3 | |
| **Women** | | | | | | | |
| Not sexually active | 781 | 14% | 0.19 | | 0 | 0.0 | |
| Married | 3,903 | 68% | 0.09 | | 53 | 45.4 | 16.5% |
| Never married | 462 | 8% | 0.11 | 9.3 (52) | 16 | 13.7 | |
| Previously married | 623 | 11% | 0.33 | | 22 | 18.8 | |
| Total | 5769 | 100% | | | 91 | 77.9 | |
| **Unions** | | | | | | | |
| SC pos. | 122 | 6% | 1.0 | | 0 | 0.0 | |
| SC neg. Man circ. | 587 | 28% | 0.0 | | 6 | 10.9 | |
| SC neg. Man uncirc. | 1220 | 59% | 0.0 | | 25 | 45.5 | |
| SD Man pos. | 81 | 4% | 0.5 | | 9 | 16.4 | |
| SD Female pos. Man circ. | 34 | 2% | 0.5 | | 3 | 5.5 | |
| SD Female pos. Man uncirc. | 32 | 2% | 0.5 | | 9 | 16.4 | |
| Total | 2076 | 100% | | | 52 | 94.7 | |

SC: sero-concordant; SD:sero-discordant; pos: HIV positive; circ: circumcised; uncirc: uncircumcised
