# Supplementary material for: The Incidence Patterns Model to Estimate the Distribution of New HIV Infections in Sub-Saharan Africa: Development and Validation of a Mathematical Model
Source: PLoS Med. 2016 Sep 13;13(9):e1002121. doi: 10.1371/journal.pmed.1002121 (PMC5021265; doi:10.1371/journal.pmed.1002121)
Supplement: S7 Table — (PDF) [file pmed.1002121.s012.pdf]

Rakai R13	Sample size	Percent	Proportion HIV +	mean duration sexual activity (variance)	Sero-conversions (SC)	Rescaled SC	ART coverage HIV+
**Men**							
Not sexually active	690	16%	0.03	7.0 (25)	0	0.0	24.8% (n=86)
Married	2,763	63%	0.12		24	27.8	
Never married circ.	291	7%	0.02		2	2.3	
Never married uncirc.	406	9%	0.03		5	5.8	
Previously married circ.	104	2%	0.24		1	1.2	
Previously married uncirc.	143	3%	0.24		9	10.4	
Total	4397	100%			41	47.5	
**Women**							
Not sexually active	838	14%	0.20	10.5 (61)	0	0.0	24.2%(n=78)
Married	3,904	67%	0.10		43	37.8	
Never married	433	7%	0.11		13	11.4	
Previously married	617	11%	0.34		18	15.8	
Total	5792	100%			74	65.1	
**Unions**							
SC pos.	158	7%	1.0		0	0.0	
SC neg. Man circ.	760	34%	0.0		7	11.5	
SC neg. Man uncirc.	1130	51%	0.0		23	37.7	
SD Man pos.	86	4%	0.5		8	13.1	
SD Female pos. Man circ.	45	2%	0.5		0	0.0	
SD Female pos. Man uncirc.	33	1%	0.5		2	3.3	
Total	2212	100%			40	65.6	

SC: sero-concordant; SD:sero-discordant; pos: HIV positive; circ: circumcised; uncirc: uncircumcised
