# Supplementary material for: The Incidence Patterns Model to Estimate the Distribution of New HIV Infections in Sub-Saharan Africa: Development and Validation of a Mathematical Model
Source: PLoS Med. 2016 Sep 13;13(9):e1002121. doi: 10.1371/journal.pmed.1002121 (PMC5021265; doi:10.1371/journal.pmed.1002121)
Supplement: S8 Table — (PDF) [file pmed.1002121.s013.pdf]

Karonga	Sample size	Percent	Proportion HIV +	mean duration sexual activity (variance)	Sero-conversions (SC)	Rescaled SC	ART coverage HIV+
**Men**							
Not sexually active	394	19%	0.00	3.4 (12)	0	0.0	
Married	1,426	67%	0.05		10	11.7	
Never married circ.	8	0%	0.00		0	0.0	
Never married uncirc.	259	12%	0.00		0	0.0	
Previously married circ.	1	0%	0.00		0	0.0	
Previously married uncirc.	33	2%	0.12		1	1.2	
Total	2121	100%			11	12.9	20% (n=308)
**Women**							
Not sexually active	287	10%	0.00	2 (14)	0	0.0	
Married	2,362	83%	0.04		14	12.2	
Never married	48	2%	0.00		1	0.9	
Previously married	157	6%	0.17		2	1.7	
Total	2854	100%			17	14.8	25% (n=529)
**Unions**							
SC pos.	20	2%	1.0		0	0.0	
SC neg. Man circ.	33	3%	0.0		0	0.0	
SC neg. Man uncirc.	995	92%	0.0		6	17.9	
SD Man pos.	26	2%	0.5		1	3.0	
SD Female pos. Man circ.	0	0%	0.5		0	0.0	
SD Female pos. Man uncirc.	10	1%	0.5		1	3.0	
Total	1084	100%			8	23.9	

SC: sero-concordant; SD:sero-discordant; pos: HIV positive; circ: circumcised; uncirc: uncircumcised
