# Supplementary material for: The Incidence Patterns Model to Estimate the Distribution of New HIV Infections in Sub-Saharan Africa: Development and Validation of a Mathematical Model
Source: PLoS Med. 2016 Sep 13;13(9):e1002121. doi: 10.1371/journal.pmed.1002121 (PMC5021265; doi:10.1371/journal.pmed.1002121)
Supplement: S9 Table — (PDF) [file pmed.1002121.s014.pdf]

Kisesa	Sample size	Percent	Proportion HIV +	mean duration sexual activity (variance)	Sero-conversions (SC)	Rescaled SC	ART coverage HIV+ (n=676)
**Men**							
Not sexually active	398	31%	0.02	3.7 (28)	0	0.0	
Married	672	53%	0.04		19	25.7	
Never married circ.	54	4%	0.00		1	1.4	
Never married uncirc.	129	10%	0.01		3	4.1	
Previously married circ.	8	1%	0.00		0	0.0	
Previously married uncirc.	11	1%	0.09		1	1.4	
Total	1272	100%			24	32.5	2.5%
**Women**							
Not sexually active	740	34%	0.05	3.0 (10)	0	0.0	
Married	1,227	56%	0.04		27	21.4	
Never married	119	5%	0.01		7	5.5	
Previously married	87	4%	0.07		16	12.7	
Total	2173	100%			50	39.6	2.5%
**Unions**							
SC pos.	29	2%	1.0		NA	NA	
SC neg. Man circ.	308	26%	0.0				
SC neg. Man uncirc.	776	64%	0.0				
SD Man pos.	59	5%	0.5				
SD Female pos. Man circ.	10	1%	0.5				
SD Female pos. Man uncirc.	26	2%	0.5				
Total	1208	100%					

SC: sero-concordant; SD:sero-discordant; pos: HIV positive; circ: circumcised; uncirc: uncircumcised
