# Supplementary material for: The Incidence Patterns Model to Estimate the Distribution of New HIV Infections in Sub-Saharan Africa: Development and Validation of a Mathematical Model
Source: PLoS Med. 2016 Sep 13;13(9):e1002121. doi: 10.1371/journal.pmed.1002121 (PMC5021265; doi:10.1371/journal.pmed.1002121)
Supplement: S10 Table — (PDF) [file pmed.1002121.s015.pdf]

		ν_m	ω_m	Υ_m	ϑ_n	ϑ_p	ν_w	ω_w	Υ_w	ρ_{10}	ρ_{11}	ρ_{13}	ρ_{14}	ρ_{12}	ρ_{15}	θ	υ	η	ϑ_c	ϑ_d	δ_{10}, δ_{11}	δ_{12}	ρ_{20}	τ_{MSM}	ρ_{18}	τ_{FSW}	ρ_{21}	τ_{MWID}	ρ_{19}	τ_{FWID}
	Northern	0.26	0.78	0.87	0.01	0.05	0.23	0.91	0.56	0.00	0.01	0.00	0.05	0.07	0.20	0.95	0.05	0.31	0.03	0.00	5.05	2.70	0.00	NA	0.58	NA	NA	NA	NA	NA
**Malawi**	Central	0.21	0.77	0.93	0.16	0.07	0.23	0.90	0.55	0.04	0.03	0.00	0.04	0.02	0.24	0.92	0.08	0.44	0.08	0.22	4.29	1.45	0.21	NA	0.75	NA	NA	NA	NA	NA
	Southern	0.21	0.73	0.90	0.37	0.41	0.20	0.85	0.50	0.02	0.03	0.42	0.42	0.12	0.43	0.90	0.10	0.47	0.41	0.50	4.17	2.71	0.21	NA	0.72	NA	NA	NA	NA	NA
	Central	0.17	0.72	0.87	0.04	0.13	0.21	0.85	0.57	0.00	0.02	0.00	0.57	0.27	0.43	0.86	0.89	0.60	0.07	0.00	5.17	3.25	NA	NA	NA	0.03	NA	NA	NA	NA
	Copperbelt	0.30	0.66	0.88	0.14	0.25	0.27	0.76	0.56	0.00	0.07	0.41	0.46	0.15	0.49	0.88	0.87	0.76	0.14	0.00	5.33	3.18	NA	NA	0.65	0.03	NA	NA	NA	NA
	Eastern	0.16	0.79	0.86	0.03	0.02	0.15	0.85	0.53	0.42	0.02	1.00	0.57	0.05	0.34	0.94	0.95	0.21	0.03	0.00	3.94	1.72	NA	NA	NA	0.03	NA	NA	NA	NA
	Luapula	0.18	0.81	0.87	0.08	0.05	0.20	0.85	0.55	0.00	0.09	1.00	0.19	0.10	0.31	0.89	0.92	0.36	0.11	0.00	3.75	3.42	NA	NA	NA	0.03	NA	NA	NA	NA
**Zambia**	Lusaka	0.26	0.64	0.85	0.11	0.07	0.28	0.75	0.67	0.24	0.07	0.00	0.42	0.13	0.45	0.82	0.80	0.35	0.12	0.00	6.67	3.83	NA	NA	NA	0.03	NA	NA	NA	NA
	Northern	0.21	0.81	0.93	0.04	0.17	0.21	0.87	0.56	0.00	0.04	0.00	0.11	0.14	0.30	0.93	0.98	0.20	0.03	0.00	4.44	2.71	NA	NA	NA	0.03	NA	NA	NA	NA
	Northwestern	0.15	0.72	0.93	0.70	0.77	0.13	0.76	0.65	0.01	0.01	0.21	0.45	0.09	0.25	0.94	0.96	0.56	0.77	0.00	4.90	3.86	NA	NA	NA	0.03	NA	NA	NA	NA
	Southern	0.21	0.69	0.88	0.02	0.06	0.17	0.75	0.68	0.00	0.05	0.00	0.49	0.15	0.36	0.88	0.90	0.27	0.05	0.00	5.22	3.62	NA	NA	NA	0.03	NA	NA	NA	NA
	Western	0.12	0.63	0.88	0.42	0.31	0.13	0.61	0.72	0.05	0.05	0.28	0.45	0.19	0.27	0.84	0.92	0.35	0.47	0.00	6.49	5.75	NA	NA	NA	0.03	NA	NA	NA	NA
	Hhohho	0.33	0.55	0.94	0.08	0.22	0.26	0.61	0.89	0.15	0.20	0.56	0.81	0.46	0.66	0.84	0.16	0.51	0.11	0.17	6.61	6.39	0.13	NA	0.61	0.02	NA	NA	NA	NA
**Swaziland**	Manzini	0.36	0.43	0.95	0.07	0.13	0.26	0.53	0.92	0.34	0.18	0.00	0.54	0.34	0.62	0.86	0.14	0.61	0.17	0.31	6.22	6.34	0.13	NA	0.61	0.02	NA	NA	NA	NA
	Shiselweni	0.44	0.38	0.94	0.08	0.21	0.29	0.48	0.91	0.11	0.13	0.30	0.70	0.40	0.58	0.82	0.18	0.49	0.12	0.12	5.87	5.92	0.13	NA	0.61	0.02	NA	NA	NA	NA
	Lubombo	0.36	0.53	0.92	0.06	0.14	0.22	0.60	0.88	0.14	0.18	0.69	0.62	0.33	0.46	0.81	0.19	0.49	0.10	0.08	6.10	5.81	0.13	NA	0.61	0.02	NA	NA	NA	NA
	Libreville-Port-Gentil	0.10	0.60	0.98	0.99	1.00	0.14	0.64	0.95	0.01	NA	0.08	NA	0.03	0.09	0.92	0.08	0.85	1.00	1.00	7.79	6.65	NA	NA	0.20	0.02	NA	NA	NA	NA
	Estuaire	0.13	0.59	0.98	0.99	1.00	0.14	0.69	0.97	0.01	NA	0.00	NA	0.05	0.18	0.94	0.06	0.60	1.00	0.88	5.86	4.68	NA	NA	0.23	0.02	NA	NA	NA	NA
	Haut-Ogooué	0.16	0.58	0.99	1.00	1.00	0.13	0.60	0.97	0.01	NA	0.00	NA	0.03	0.40	0.92	0.08	0.77	0.99	1.00	6.43	4.48	NA	NA	NA	NA	NA	NA	NA	NA
	Moyen-Ogooué	0.13	0.51	1.00	1.00	NA	0.14	0.66	0.99	0.03	NA	NA	NA	0.05	0.47	0.91	0.09	0.64	0.99	1.00	8.17	7.61	NA	NA	NA	NA	NA	NA	NA	NA
**Gabon**	Ngounié	0.13	0.59	0.97	1.00	1.00	0.14	0.66	0.91	0.02	NA	0.00	NA	0.06	0.28	0.87	0.13	0.49	1.00	1.00	5.02	4.98	NA	NA	NA	NA	NA	NA	NA	NA
	Nyanga	0.16	0.59	0.99	1.00	1.00	0.12	0.72	0.94	0.01	NA	0.11	NA	0.04	0.37	0.95	0.05	0.67	1.00	1.00	6.87	5.02	NA	NA	NA	NA	NA	NA	NA	NA
	Ogooué Maritime	0.11	0.71	0.95	1.00	1.00	0.13	0.78	0.97	0.01	NA	0.10	NA	0.04	0.00	0.94	0.06	0.54	1.00	1.00	5.34	6.37	NA	NA	NA	NA	NA	NA	NA	NA
	Ogooué-Ivindo	0.11	0.63	0.95	1.00	1.00	0.09	0.67	0.93	0.00	NA	0.14	NA	0.02	0.00	0.94	0.06	0.40	0.99	1.00	6.30	4.28	NA	NA	NA	NA	NA	NA	NA	NA
	Ogooué-Lolo	0.15	0.59	0.94	1.00	1.00	0.14	0.70	0.95	0.00	NA	0.00	NA	0.03	0.12	0.93	0.07	0.58	1.00	1.00	6.10	5.49	NA	NA	NA	NA	NA	NA	NA	NA
	Woleu-Ntem	0.09	0.71	0.97	1.00	1.00	0.09	0.75	0.92	0.01	NA	0.47	NA	0.07	0.21	0.91	0.09	0.58	1.00	1.00	5.42	7.87	NA	NA	NA	NA	NA	NA	NA	NA
	Kigali City	0.37	0.71	0.93	0.37	0.55	0.44	0.80	0.69	0.00	0.07	0.00	0.16	0.11	0.16	0.94	0.91	0.74	0.35	0.40	5.84	3.89	NA	0.00	0.56	0.02	NA	0.00	NA	NA
	South	0.40	0.86	0.91	0.09	0.08	0.42	0.84	0.55	0.00	0.00	1.00	0.28	0.12	0.11	0.98	0.99	0.00	0.05	0.00	4.20	4.80	NA	0.00	0.55	0.01	NA	0.00	NA	NA
**Rwanda**	West	0.40	0.88	0.94	0.38	0.19	0.43	0.88	0.58	0.03	0.00	0.00	0.15	0.04	0.15	0.98	0.97	0.39	0.17	0.00	3.54	3.99	NA	0.00	0.55	0.01	NA	0.00	NA	NA
	North	0.38	0.86	0.91	0.22	0.00	0.43	0.88	0.55	0.00	0.00	NA	0.00	0.04	0.11	0.98	0.98	0.00	0.03	0.00	4.16	5.40	NA	0.00	0.47	0.01	NA	0.00	NA	NA
	East	0.35	0.83	0.82	0.18	0.19	0.38	0.84	0.49	0.00	0.02	0.00	0.04	0.01	0.07	0.98	0.99	0.49	0.10	0.25	5.12	3.28	NA	0.00	0.33	0.01	NA	0.00	NA	NA
	Nairobi	0.09	0.60	0.97	0.89	0.96	0.23	0.68	0.90	0.02	0.00	0.09	1.00	0.06	0.81	0.89	0.96	0.90	0.93	0.90	7.68	5.65	0.13	0.01	0.49	0.03	0.19	2.50E-03	1.85E-01	5.00E-04
	Central	0.24	0.64	0.99	0.99	1.00	0.26	0.84	0.74	0.00	0.52	0.00	NA	0.01	0.34	0.97	0.99	0.54	0.99	1.00	6.62	6.43	0.15	0.01	0.46	0.02	0.19	2.50E-05	1.85E-01	5.00E-06
	Coast	0.18	0.77	0.92	0.98	0.90	0.22	0.85	0.80	0.00	0.00	0.00	0.00	0.07	0.26	0.94	0.99	0.76	0.98	1.00	6.21	5.56	0.16	0.01	0.35	0.02	0.31	2.50E-03	3.10E-01	5.00E-04
**Kenya**	Eastern	0.33	0.68	0.90	0.99	0.86	0.26	0.85	0.74	0.01	0.00	0.00	1.00	0.07	0.11	0.97	0.97	0.56	0.96	1.00	7.36	4.99	0.15	0.01	0.46	0.02	0.19	2.50E-04	1.85E-01	5.00E-06
	Nyanza	0.21	0.64	0.98	0.42	0.43	0.19	0.74	0.64	0.01	0.06	0.00	0.47	0.03	0.65	0.85	0.91	0.29	0.60	0.37	4.40	2.12	0.15	0.01	0.54	0.02	0.19	2.50E-05	1.85E-01	5.00E-06
	Rift_Valley	0.20	0.72	0.98	0.88	0.97	0.27	0.79	0.85	0.00	0.15	0.00	0.00	0.11	0.81	0.97	0.98	0.66	0.96	0.81	6.76	6.70	0.15	0.01	0.46	0.02	0.19	2.50E-05	1.85E-01	5.00E-06
	Western	0.30	0.64	0.94	0.95	0.82	0.32	0.84	0.77	0.03	0.06	0.00	1.00	0.04	0.46	0.96	0.97	0.70	0.93	1.00	6.63	3.20	0.15	0.01	0.54	0.02	0.19	2.50E-05	1.85E-01	5.00E-06
	Northeastern	0.36	0.96	0.91	0.81	1.00	0.29	0.96	1.00	0.00	NA	0.00	NA	NA	0.00	0.98	0.99	1.00	1.00	1.00	5.67	NA	0.15	0.01	0.46	0.02	0.19	2.50E-05	1.85E-01	5.00E-06

S10 Table. Data for Gabon, Kenya, Malawi, Rwanda, Swaziland and Zambia by province
