# Supplementary material for: The Incidence Patterns Model to Estimate the Distribution of New HIV Infections in Sub-Saharan Africa: Development and Validation of a Mathematical Model
Source: PLoS Med. 2016 Sep 13;13(9):e1002121. doi: 10.1371/journal.pmed.1002121 (PMC5021265; doi:10.1371/journal.pmed.1002121)
Supplement: S11 Table — (PDF) [file pmed.1002121.s016.pdf]

	Sample size	ν_m	ν_w	ρ_{10}	ρ_{11}	ρ_{13}	ρ_{14}	ρ_{12}	ρ_{15}	θ	υ	η	ϑ_c	ϑ_d	ω_m	Υ_m	ϑ_n	ϑ_p	ω_w	Υ_w	ρ_{20}	τ_{MSM}	ρ_{18}	τ_{FSW}	ρ_{21}	τ_{MWID}	ρ_{19}	τ_{FWID}
**Malawi**	Northern	1304	4189	4	166	3	19	62	51	566	530	36	12	507	968	233	181	23	3252	456	NA	NA	43	NA	NA	NA	NA	NA
	Central	2611	7862	69	348	4	24	105	105	1293	1203	88	39	1160	2063	513	449	30	6113	825	201	NA	64	NA	NA	NA	NA	NA
	Southern	3284	10969	173	369	22	33	227	232	1485	1335	148	61	1185	2585	693	595	60	8764	1623	201	NA	166	NA	NA	NA	NA	NA
**Zambia**	Central	600	672	4	94	2	14	32	32	205	174	30	18	155	492	146	123	18	522	97	NA	NA	NA	400	NA	NA	NA	NA
	Copperbelt	812	829	16	98	3	15	62	45	187	166	21	15	146	584	201	162	25	611	142	NA	NA	283	400	NA	NA	NA	NA
	Eastern	857	940	3	104	1	16	68	52	398	372	26	7	348	712	157	131	21	777	152	NA	NA	NA	400	NA	NA	NA	NA
	Luapula	560	704	5	62	1	9	45	35	236	211	25	9	188	457	93	77	11	556	97	NA	NA	NA	400	NA	NA	NA	NA
	Lusaka	962	939	14	162	2	26	84	39	301	251	50	18	204	722	254	215	36	691	172	NA	NA	NA	400	NA	NA	NA	NA
	Northern	715	783	5	81	1	7	44	33	285	263	22	4	252	554	127	103	9	608	108	NA	NA	NA	400	NA	NA	NA	NA
	Northwestern	630	685	81	42	6	3	68	39	207	192	15	9	181	531	170	150	11	590	172	NA	NA	NA	400	NA	NA	NA	NA
	Southern	774	822	1	140	1	19	106	48	271	235	36	11	210	607	197	168	23	682	191	NA	NA	NA	400	NA	NA	NA	NA
	Western	590	772	56	88	5	13	159	56	211	171	40	18	152	512	213	172	21	662	278	NA	NA	NA	400	NA	NA	NA	NA
**Swaziland**	Hhohho	1019	1263	22	219	2	10	294	39	186	155	31	17	102	683	322	278	15	944	386	321	NA	320	400	NA	NA	NA	NA
	Manzini	1186	1475	25	334	1	12	431	37	185	159	26	14	109	766	443	404	19	1102	537	321	NA	320	400	NA	NA	NA	NA
	Shiselweni	838	1083	17	221	3	14	339	32	112	94	18	9	61	496	305	268	17	788	415	321	NA	320	400	NA	NA	NA	NA
	Lubombo	1113	1166	17	258	3	20	296	42	176	141	35	19	91	771	368	321	23	908	381	321	NA	320	400	NA	NA	NA	NA
**Gabon**	Libreville-Port-Gentil	989	1557	302	2	5	0	239	15	329	304	25	21	299	896	400	326	5	1361	557	NA	NA	423	500	NA	NA	NA	NA
	Estuaire	617	844	189	2	6	0	132	6	170	157	13	8	156	543	242	205	6	740	277	NA	NA	601	500	NA	NA	NA	NA
	Haut-Ogooué	508	875	163	1	3	0	174	5	183	170	13	10	167	436	190	168	3	775	354	NA	NA	NA	NA	NA	NA	NA	NA
	Moyen-Ogooué	436	681	164	0	0	0	117	3	135	123	12	8	120	389	196	164	0	594	237	NA	NA	NA	NA	NA	NA	NA	NA
	Ngounié	565	871	170	0	9	0	127	12	206	186	20	10	184	495	209	171	9	772	304	NA	NA	NA	NA	NA	NA	NA	NA
	Nyanga	455	672	135	0	3	0	94	6	149	142	7	4	141	392	153	137	3	608	191	NA	NA	NA	NA	NA	NA	NA	NA
	Ogooué Maritime	449	560	91	0	5	0	60	3	163	153	10	6	151	406	142	93	5	501	153	NA	NA	NA	NA	NA	NA	NA	NA
	Ogooué-Ivindo	612	984	171	0	9	0	160	9	230	218	11	5	218	542	210	173	9	896	317	NA	NA	NA	NA	NA	NA	NA	NA
	Ogooué-Lolo	563	741	163	0	12	0	106	6	177	165	11	5	161	488	207	165	12	649	234	NA	NA	NA	NA	NA	NA	NA	NA
	Woleu-Ntem	460	637	114	0	5	0	85	11	141	127	14	9	124	420	143	117	5	581	160	NA	NA	NA	NA	NA	NA	NA	NA
**Rwanda**	Kigali City	932	1890	61	101	7	6	92	36	329	308	21	14	281	591	186	167	13	1063	242	NA	100	233	100	NA	100	NA	100
	South	1517	3340	9	102	1	9	88	65	678	668	10	0	662	915	133	111	11	1956	331	NA	100	245	100	NA	100	NA	100
	West	1413	3138	37	62	1	5	68	48	643	632	11	4	615	858	111	100	6	1804	237	NA	100	251	100	NA	100	NA	100
	North	971	2199	16	58	0	7	47	45	449	443	6	0	436	601	83	74	7	1262	163	NA	100	260	100	NA	100	NA	100
	East	1496	3104	25	112	5	24	71	79	709	693	16	8	683	970	175	137	29	1920	328	NA	100	123	100	NA	100	NA	100
**Kenya**	Nairobi	428	952	98	19	4	2	207	6	141	128	13	11	123	388	145	129	5	724	258	2685	100	910	100	729.6	100	182.4	100
	Central	394	973	85	2	2	2	248	2	112	109	3	1	108	302	132	103	1	720	158	2437.5	100	1529	100	10	100	10	100
	Coast	456	1149	79	4	9	2	316	2	160	151	9	6	149	369	109	89	9	896	186	2190	100	3143	100	110.4	100	27.6	100
	Eastern	457	1127	72	2	6	2	321	5	161	158	3	1	156	315	106	82	7	839	153	2437.5	100	1529	100	10	100	10	100
	Nyanza	544	1318	50	71	3	2	380	6	215	181	34	13	158	431	143	129	4	1073	278	2437.5	100	534	100	10	100	10	100
	Rift_Valley	549	1278	98	20	4	2	326	3	207	196	11	6	192	447	150	129	4	955	218	2437.5	100	1529	100	10	100	10	100
	Western	457	1039	88	6	3	2	281	2	160	155	5	2	149	342	124	99	3	764	169	2437.5	100	534	100	10	100	10	100
	Northeastern	230	608	4	2	2	2	168	0	72	71	1	1	70	146	20	8	1	424	14	2437.5	100	1529	100	10	100	10	100

S11 Table. Sample sizes of the data for Gabon, Kenya, Malawi, Rwanda, Swaziland and Zambia by province.
