# Supplementary material for: The Incidence Patterns Model to Estimate the Distribution of New HIV Infections in Sub-Saharan Africa: Development and Validation of a Mathematical Model
Source: PLoS Med. 2016 Sep 13;13(9):e1002121. doi: 10.1371/journal.pmed.1002121 (PMC5021265; doi:10.1371/journal.pmed.1002121)
Supplement: S12 Table — (PDF) [file pmed.1002121.s017.pdf]

**Recent Pop Survey w HIV**	Last	Survey
Benin	2009	2012
Botswana		2014
Burkina Faso	2009	2010
Burundi	2014	2010
Cameroon		2011
Central African Republic		2010
Cote d'Ivoire	2009	2012
Ethiopia		2011
Gabon		2012
Gambia		2013
Guinea		2012
Kenya	2005	2012
Malawi	2008	2010
Namibia		2013
Niger		2012
Nigeria	2009	2011
Rwanda		2010
Senegal	2009	2011
South Africa		2012
Swaziland	2008	2011
Tanzania		2012
Togo		2013
Uganda	2008	2011
Zambia	2008	2013
Zimbabwe	2010	2012
**Older Pop Survey with HIV**	Last	Survey
Congo		2009
DRC		2007
Lesotho	2008	2009
Liberia		2007
Mozambique		2009
Sierra Leone	2010	2008
**No pop survey w HIV testing**	Last	Survey
Angola	2012	NA
Chad		NA
Djibouti		NA
Equatorial Guinea		NA
Eritrea	2015	NA
Ghana	2014	NA
Guinea Bissau		NA
Mali		NA
Somalia		NA
South Sudan	2013	NA
Sudan		NA

S12 Table. Latest demographic health surveys implemented in Sub-Saharan African countries.
