# Supplementary material for: The Incidence Patterns Model to Estimate the Distribution of New HIV Infections in Sub-Saharan Africa: Development and Validation of a Mathematical Model
Source: PLoS Med. 2016 Sep 13;13(9):e1002121. doi: 10.1371/journal.pmed.1002121 (PMC5021265; doi:10.1371/journal.pmed.1002121)
Supplement: S13 Table — (PDF) [file pmed.1002121.s018.pdf]

Symbol	Parameter definition	Rwanda	Gabon	Zambia	Kenya	Malawi	Swaziland	Distribution	Sample Size	Reference
**Mixing matrix**										
	**Proportion of extramarital partnerships among:**									
χ_1	married men	0.15	0.18	0.15	0.15	0.15	0.2	Beta	100	Based on proportion of married men and women reporting extra-marital sex in DHS
χ_2	married women	0.1	0.1	0.1	0.1	0.1	0.1	Beta	100	
χ_3	men that are with married women	0.5	0.6	0.8	0.8	0.8	0.2	Beta	100	Assumption
χ_4	women that are with married men	0.95	0.9	0.85	0.5	0.65	0.9	Beta	100	Assumption
χ_5	men that are not with other married women that are with never married women	0.3	0.1	0.2	0.1	0.2	0.5	Beta	100	Assumption
χ_6	women that are not with other married men that are with never married men	0.3	0.2	0.2	0.8	0.9	0.3	Beta	100	Assumption
χ_7	men that are not with other married women and that are not with never married women that are with previously married women	0.1	0.2	0.1	0.1	0.6	0.2	Beta	100	Assumption
χ_8	women that are not with other married men and that are not with never married men that are with previously married men	0.7	0.3	0.25	0.3	0.5	0.5	Beta	100	Assumption
χ_9	men that are not with previously married women that are with FSW	0.999	0.999	0.999	0.999	0.999	0.999	Beta	100	Based on proportion of married men reporting paying for sex in the DHS
χ_{10}	women that are not with previously married men that are with MSM	0.7	0.7	0.7	0.7	0.7	0.7	Beta	100	Assumption
	**Proportion of dissassortative partnerships among:**									
χ_{11}	never married women	0.1	0.2	0.1	0.1	0.1	0.2	Beta	100	Based on proportion of 15-24 year old women reporting having sex with partners 10+ years older than themselves in DHS
χ_{12}	never married men	0.1	0.6	0.2	0.6	0.4	0.2	Beta	100	Assumption
χ_{13}	never married men that are with married women	0.3	0.3	0.3	0.3	0.3	0.3	Beta	100	Assumption
χ_{14}	never married women that are with married men	0.8	0.5	0.8	0.85	0.9	0.9	Beta	100	Assumption
χ_{15}	never married women that are with previously married men	0.8	0.5	0.8	0.9	0.9	0.9	Beta	100	Assumption
χ_{16}	never married men that are with previously married women	0.5	0.5	0.5	0.3	0.3	0.4	Beta	100	
χ_{17}	never married men that are not with previously married women that are with FSW	0.9999	0.9999	0.9999	0.9999	0.9999	0.9999	Beta	100	Based on proportion of men 15-24 who report paying for sex in DHS
χ_{18}	never married women that are not with previously married men that are with MSM	0.9	0.9	0.9	0.9	0.9	0.9	Beta	100	Assumption

S13 Table. Priors for the parameters informing the transmission model

Symbol	Parameter definition	Rwanda	Gabon	Zambia	Kenya	Malawi	Swaziland	Distribution	Sample Size	Reference
**Mixing matrix**										
χ_{19}	previously married women that are not with previously married men	0.5	0.4	0.8	0.6	0.7	0.6	Beta	100	Assumption
χ_{20}	previously married men that are not with previously married women	0.9	0.1	0.3	0.1	0.1	0.1	Beta	100	Assumption
χ_{21}	previously married women that are with married men	0.85	0.8	0.8	0.6	0.7	0.95	Beta	100	Assumption
χ_{22}	previously married men that are with married women	0.6	0.6	0.6	0.7	0.7	0.6	Beta	100	Assumption
χ_{23}	previously married men that are not with married women that are with never married women	0.6	0.6	0.6	0.6	0.6	0.6	Beta	100	Assumption
χ_{24}	previously married women that are not with married men that are with never married men	0.9	0.85	0.85	0.85	0.85	0.85	Beta	100	Assumption
χ_{25}	previously married men that are not with never married women that are with FSW	0.999	0.999	0.999	0.999	0.999	0.999	Beta	100	Assumption based on proportion of previously married men reporting paying for sex in the DHS
χ_{26}	previously married women that are not with never married men that are with MSM	0.9	0.9	0.9	0.9	0.9	0.9	Beta	100	Assumption
χ_{27}	MWID	0.3	0.3	0.3	0.1	0.1	0.2	Beta	100	Based on the fact that mixing among PWID is highly assortative
χ_{28}	among MWID that are with FWID	0.7	0.7	0.7	0.7	0.7	0.7	Beta	100	Based on the fact that mixing among PWID is highly assortative
χ_{29}	MWID that are not with FWID that are with married women	0.1	0.1	0.1	0.1	0.1	0.1	Beta	100	Assumption
χ_{30}	MWID that are not with FWID that are not with married women that are with never married women	0.2	0.2	0.2	0.2	0.2	0.2	Beta	100	Assumption
χ_{31}	MWID that are not with FWID that are not with married women that are not with never married women that are with previously married women	0.3	0.3	0.3	0.3	0.3	0.3	Beta	100	Assumption
χ_{32}	MWID that are not with FWID that are not with married women that are not with never married women that are not with previously married women that are with FSW	0.8	0.8	0.8	0.8	0.8	0.8	Beta	100	Assumption
χ_{33}	FWID	0.5	0.5	0.5	0.8	0.8	0.7	Beta	100	Based on the fact that mixing among PWID is very assortative
χ_{34}	FWID that are with MWID	0.8	0.8	0.8	0.8	0.8	0.8	Beta	100	Based on the fact that mixing among PWID is very assortative
χ_{35}	FWID that are not with MWID that are with married men	0.2	0.2	0.2	0.2	0.2	0.2	Beta	100	Assumption
χ_{36}	FWID that are not with MWID that are not with married men that are with never married men	0.5	0.5	0.5	0.5	0.5	0.5	Beta	100	Assumption
χ_{37}	FWID that are not with MWID that are not with married men that are not with never married men that are with previously married men	0.5	0.5	0.5	0.5	0.5	0.5	Beta	100	Assumption

S13 Table ctd. Priors for the parameters informing the transmission model

Symbol	Parameter definition	Rwanda	Gabon	Zambia	Kenya	Malawi	Swaziland	Distribution	Sample Size	Reference
**Mixing Matrix**										
χ_{38}	MSM	0.1	0.15	0.15	0.1	0.1	0.1	Beta	100	Based on the fact that mixing among MSM is very assortative
χ_{39}	MSM that are with married women	0.3	0.3	0.3	0.3	0.3	0.3	Beta	100	Based on proportion of MSM who report being married
χ_{40}	MSM that are not with married women that are with never married women	0.3	0.3	0.3	0.2	0.2	0.3	Beta	100	Assumption
χ_{41}	MSM that are not with married women that are not with never married women that are with previously married women	0.2	0.2	0.2	0.2	0.2	0.2	Beta	100	Assumption
χ_{42}	MSM that are not with married women that are not with never married women that are not with previously married women that are with MWID	0.02	0.02	0.02	0.02	0.02	0.02	Beta	100	Assumption
χ_{43}	MSM that are not with married women that are not with never married women that are not with previously married women that are not with MWID that are with FSW	0.9	0.9	0.9	0.8	0.8	0.8	Beta	100	Assumption
χ_{44}	FSW that are not with never married men	0.1	0.1	0.1	0.1	0.1	0.1	Beta	100	Based on the fact that the majority of men reporting paying for sex are married men
χ_{45}	FSW that are not with never married men that are with married men	0.9	0.8	0.9	0.9	0.9	0.8	Beta	100	Assumption
χ_{46}	FSW that are not with never married men that are not with married men that are with previously married men	0.95	0.95	0.95	0.95	0.95	0.95	Beta	100	Assumption
χ_{47}	FSW that are not with never married men that are not with married men that are not with previously married men that are with MWID	0.2	0.2	0.2	0.2	0.2	0.2	Beta	100	Assumption
	**Transmissibility degree among:**							**Distribution**	**Standard deviation**	
$T_{1\text{-}4}$	Serodiscordant positive unions where the woman is positive	0.1	0.1	0.1	0.1	0.1	0.1	Normal	0.02	Baseline
$T_{5\text{-}8}$	serodiscordant positive unions where the man is positive	0.2	0.2	0.2	0.2	0.2	0.2	Normal	0.04	2 folds higher transmission probability compared to women
T_9	Seroconconrdant positive unions	0.15	0.15	0.15	0.15	0.15	0.15	Normal	0.03	Average between transmissibility in serodiscordant unions where man and woman is positive respectively
$T_{10\text{-}11}$	Never married men	0.16	0.16	0.16	0.16	0.16	0.16	Normal	0.032	Higher condom use than among married men and two folds transmission probability compared to women
T_{12}	Never married women	0.08	0.08	0.08	0.08	0.08	0.08	Normal	0.016	Higher condom use than among married women
$T_{13\text{-}14}$	Previously married men	0.2	0.2	0.2	0.2	0.2	0.2	Normal	0.04	Assumed to be equal to that among married men
T_{15}	Previously married women	0.1	0.1	0.1	0.1	0.1	0.1	Normal	0.02	Assumed to be equal to that among married women
T_{18}	FSW	0.1	0.1	0.1	0.1	0.1	0.1	Normal	0.02	Assumed to be equal to that among married women (fewer sex acts/higher condom use)
T_{19}	FWID	0.2	0.2	0.2	0.2	0.2	0.2	Normal	0.04	Assumed to be higher than among married women
T_{20}	MSM	0.3	0.3	0.3	0.3	0.3	0.3	Normal	0.06	Assumed to be higher than among married men (anal sex)
T_{21}	MWID	0.2	0.2	0.2	0.2	0.2	0.2	Normal	0.04	Assumed to be equal to that among married men

S13 Table ctd. Priors for the parameters informing the transmission model
