# Supplementary material for: The Incidence Patterns Model to Estimate the Distribution of New HIV Infections in Sub-Saharan Africa: Development and Validation of a Mathematical Model
Source: PLoS Med. 2016 Sep 13;13(9):e1002121. doi: 10.1371/journal.pmed.1002121 (PMC5021265; doi:10.1371/journal.pmed.1002121)
Supplement: S1 Text — (PDF) [file pmed.1002121.s019.pdf]

Supporting material

The Incidence Patterns Model to estimate the distribution of new HIV infections in sub-Saharan Africa: development and validation of a mathematical model

Annick Bórquez, Anne Cori, Erica L. Pufall, Jingo Kasule, Emma Slaymaker, Alison Price, Jocelyn Elmes, Basia Zaba, Amelia C. Crampin, Joseph Kagaayi, Tom Lutalo, Mark Urassa, Simon Gregson, Timothy B. Hallett.

CONTENTS

1. Incidence Patterns Model (IPM) specifications 2
   1.1. Disaggregation of the population 2
   1.2. Model equations 2
   1.3. Model fitting 6
2. Model validation 8
   2.1. Description of ALPHA Network studies 8
   2.2. Data analysis of incidence by risk determinants 8
   2.3. Implementation of model validation 9
3. Application in countries 10
4. Transmission module 11

1. Incidence Patterns Model (IPM) specifications

1.1. Disaggregation of the population

The model is a deterministic compartmental model which produces estimates of the distribution of infections acquired by group and province over a single time step (1 year). The population is divided into 21 groups: unions (which are disaggregated into 9 groups depending on sero-status, gender of the infected partner, circumcision status and ART status), never married men (disaggregated into 2 groups depending on circumcision status), never married women, previously married men (disaggregated into 2 groups depending on circumcision status), previously married women, men and women not sexually active in the past 12 months, Female Sex Workers (FSW), Men who Have Sex with Men (MSM), People Who Inject Drugs (PWID) (divided into two groups depending on gender) and the model is applied to each province in the country. A full description of the groups, including indices, characteristics and definition is available in Table 2 in the main text and described graphically in S1 Fig.

1.2. Model equations

Data on the distribution of the population excluding key populations is obtained from the DHS surveys which collect the information into three main datasets: unions, men and women described in the model by $C_{i,r}$, $M_{i,r}$ and $W_{i,r}$ respectively corresponding to the proportion in sub-group i (characterised by different determinants of infection) and administrative division (or province) r. The different risk determinant categories for each group are described in S1 Table.

The equations for the distribution of people in each group are below (eq. 1 to 16), where Θ is the proportion of unions that are sero-concordant for HIV (positive and negative), η is the proportion of sero-discordant unions where the man is negative, υ is the proportion of sero-concordant unions that are negative, ϑ_d and ϑ_c are the proportion of negative men in sero-discordant and sero-concordant unions respectively who are circumcised. For men (M) and women (W) ν is the proportion who have not been sexually active in the past 12 months, ω is the proportion sexually active who are married/cohabiting, Υ is the proportion sexually active who never married, ϑ_n and ϑ_p are the proportion of never and previously married men respectively who are circumcised. All these parameters are province specific as indicated by the subscript r.

$$C_{1,r} = (1 - \theta_r) \cdot \eta_r \cdot \vartheta_{d,r}$$ ...1

$$C_{2,r} = (1 - \theta_r) \cdot \eta_r \cdot (1 - \vartheta_{d,r})$$ ...2

$$C_{3,r} = (1 - \theta_r) \cdot (1 - \eta_r)$$ ...3

$$C_{4,r} = \theta_r \cdot \upsilon_r \cdot \vartheta_{c,r}$$ ...4

$$C_{5,r} = \theta_r \cdot \upsilon_r \cdot (1 - \vartheta_{c,r})$$ ...5

$$C_{6,r} = \theta_r \cdot (1 - \upsilon_r)$$ ...6

$$M_{1,r} = (1 - \nu_{M,r}) \cdot \omega_{M,r}$$ ...7

$$M_{2,r} = (1 - \nu_{M,r}) \cdot (1 - \omega_{M,r}) \cdot \gamma_{M,r} \cdot \vartheta_{n,r}$$ ...8

$$M_{3,r} = (1 - \nu_{M,r}) \cdot (1 - \omega_{M,r}) \cdot \gamma_{M,r} \cdot (1 - \vartheta_{n,r})$$ ...9

$$M_{4,r} = (1 - \nu_{M,r}) \cdot (1 - \omega_{M,r}) \cdot (1 - \gamma_{M,r}) \cdot \vartheta_{p,r}$$ ...10

$$M_{5,r} = (1 - \nu_{M,r}) \cdot (1 - \omega_{M,r}) \cdot (1 - \gamma_{M,r}) \cdot (1 - \vartheta_{p,r})$$ ...11

$$M_{6,r} = \nu_{M,r}$$ ...12

$$W_{1,r} = (1 - \nu_{W,r}) \cdot \omega_{W,r}$$ ...13

$$W_{2,r} = (1 - \nu_{W,r}) \cdot (1 - \omega_{W,r}) \cdot \gamma_{W,r}$$ ...14

$$W_{4,r} = (1 - \nu_{W,r}) \cdot (1 - \omega_{W,r}) \cdot (1 - \gamma_{W,r})$$ ...15

$$W_{6,r} = \nu_{W,r}$$ ...16

These terms are used in the model to calculate the distribution of the total population κ including key populations and disaggregation by ART status for sero-discordant unions, as shown in equations 17 to 37. The subscripts correspond to the groups described in table S1. $\tau_{MSM,r}$, $\tau_{FSW,r}$, $\tau_{MWID,r}$, $\tau_{FWID,r}$ correspond to the proportion of men and women who are MSM, FSW and PWID respectively in each province. ζ_r corresponds to the ART coverage among HIV positive unions in each province. It is assumed to be constant across groups as currently available data on ART are rarely disaggregated.

$$\kappa_{1,r} = (M_{1,r} \cdot (1 - \tau_{MSM,r} - \tau_{MWID,r}) + W_{1,r} \cdot (1 - \tau_{FSW,r} - \tau_{FWID,r}))/2 \cdot C_{1,r} \cdot (1 - \zeta_r) \quad ...17$$

$$\kappa_{2,r} = (M_{1,r} \cdot (1 - \tau_{MSM,r} - \tau_{MWID,r}) + W_{1,r} \cdot (1 - \tau_{FSW,r} - \tau_{FWID,r}))/2 \cdot C_{1,r} \cdot \zeta_r \quad ...18$$

$$\kappa_{3,r} = (M_{1,r} \cdot (1 - \tau_{MSM,r} - \tau_{MWID,r}) + W_{1,r} \cdot (1 - \tau_{FSW,r} - \tau_{FWID,r}))/2 \cdot C_{2,r} \cdot (1 - \zeta_r) \quad ...19$$

$$\kappa_{4,r} = (M_{1,r} \cdot (1 - \tau_{MSM,r} - \tau_{MWID,r}) + W_{1,r} \cdot (1 - \tau_{FSW,r} - \tau_{FWID,r}))/2 \cdot C_{2,r} \cdot \zeta_r \quad ...20$$

$$\kappa_{5,r} = (M_{1,r} \cdot (1 - \tau_{MSM,r} - \tau_{MWID,r}) + W_{1,r} \cdot (1 - \tau_{FSW,r} - \tau_{FWID,r}))/2 \cdot C_{3,r} \cdot (1 - \zeta_r) \quad ...21$$

$$\kappa_{6,r} = (M_{1,r} \cdot (1 - \tau_{MSM,r} - \tau_{MWID,r}) + W_{1,r} \cdot (1 - \tau_{FSW,r} - \tau_{FWID,r}))/2 \cdot C_{3,r} \cdot \zeta_r \quad ...22$$

$$\kappa_{7,r} = (M_{1,r} \cdot (1 - \tau_{MSM,r} - \tau_{MWID,r}) + W_{1,r} \cdot (1 - \tau_{FSW,r} - \tau_{FWID,r}))/2 \cdot C_{4,r} \quad ...23$$

$$\kappa_{8,r} = (M_{1,r} \cdot (1 - \tau_{MSM,r} - \tau_{MWID,r}) + W_{1,r} \cdot (1 - \tau_{FSW,r} - \tau_{FWID,r}))/2 \cdot C_{5,r} \quad ...24$$

$$\kappa_{9,r} = (M_{1,r} \cdot (1 - \tau_{MSM,r} - \tau_{MWID,r}) + W_{1,r} \cdot (1 - \tau_{FSW,r} - \tau_{FWID,r}))/2 \cdot C_{6,r} \quad ...25$$

$$\kappa_{10,r} = M_{2,r} \cdot (1 - \tau_{MSM,r} - \tau_{MWID,r})/2 \quad ...26$$

$$\kappa_{11,r} = M_{3,r} \cdot (1 - \tau_{MSM,r} - \tau_{MWID,r})/2 \quad ...27$$

$$\kappa_{12,r} = W_{2,r} \cdot (1 - \tau_{FSW,r} - \tau_{FWID,r})/2 \quad ...28$$

$$\kappa_{13,r} = M_{4,r} \cdot (1 - \tau_{MSM,r} - \tau_{MWID,r})/2 \quad ...29$$

$$\kappa_{14,r} = M_{5,r} \cdot (1 - \tau_{MSM,r} - \tau_{MWID,r})/2 \quad ...30$$

$$\kappa_{15,r} = W_{3,r} \cdot (1 - \tau_{FSW,r} - \tau_{FWID})/2 \quad ...31$$

$$\kappa_{16,r} = M_{6,r} \cdot (1 - \tau_{MSM,r} - \tau_{MWID,r})/2 \quad ...32$$

$$\kappa_{17,r} = W_{4,r} \cdot (1 - \tau_{FSW,r} - \tau_{FWID,r})/2 \quad ...33$$

$$\kappa_{18,r} = \tau_{FSW,r}/2 \quad ...34$$

$$\kappa_{19,r} = \tau_{FWID,r}/2 \quad ...35$$

$$\kappa_{20,r} = \tau_{MSM,r}/2 \quad ...36$$

$$\kappa_{21,r} = \tau_{MWID,r}/2 \quad ...37$$

Incidence in each group is calculated by applying an infection hazard to the susceptible population based on group specific estimates of incidence from large trials or on incidence inference methods from prevalence and duration of exposure. The force of infection λ in each group i and province r is calculated in equations 38 to 58. ξ_S and ξ_e are the transmission hazard per 100 person years at risk when exposed to stable and external partners respectively, φ is the relative risk of infection among circumcised men and o is the relative risk of infection among people whose HIV positive partner is on ART, $\rho_{i,r}$ is the HIV prevalence in each group and province , $\varsigma_{13\text{-}14}$ and ς_{15} are the relative risk of infection among previously married men and women respectively as compared to their married counterparts. $\delta_{10\text{-}11,r}$ and $\delta_{12,r}$ are the duration of sexual activity among never married men and women respectively, $\delta_{18,r}$, $\delta_{19,r}$, $\delta_{20,r}$ and $\delta_{21,r}$ are the duration of stay in the FSW, FWID, MSM and MWID groups respectively which incorporates the average duration of risk practice, the average duration of infection depending on ART and the average life expectancy.

Groups 1 and 2 are sero-discordant unions where the woman is HIV positive and the man is circumcised. The risk of transmission comes from within the partnership (ξ_S) and from external partnerships (ξ_e) and it is reduced by the effect of circumcision (φ). In group 2 the risk of transmission from the stable partner is also reduced by the effect of ART (o).
Group 3 and 4 are also sero-discordant unions where the woman is positive but the man is not circumcised. In group 4, transmission from the stable partner is reduced by the effect of ART (o).

Group 5 and 6 are sero-discordant unions where the man is positive and therefore circumcision is not represented. In group 6, transmission from the stable partner is reduced by the effect of ART (o).

Group 7 and 8 are sero-concordant negative unions and therefore they can only get infected through external partnerships (ξ_e). In group 7 the man is circumcised but since in this case the infection hazard applies to both men and women and the circumcision effect only protects men, an adjustment is made.

Group 9 are sero-concordant positive unions and, although they are at risk of re-infection, the FOI is set at zero because this would not contribute to the number of new infection.

Groups 10 and 11 are circumcised and not circumcised never married men respectively. The FOI is a function of their HIV prevalence ($\rho_{i,r}$) and duration of sexual activity ($\delta_{10\text{-}11.r}$).

The FOI among never married women in group 12 is calculated following the same method.

Groups 13 to 15 are previously married men (13 and 14) and women (15) whose force of infection is estimated as being a factor (ς) of the transmission hazard from both stable and external partnerships.

Groups 16 and 17 are not sexually active men and women respectively and therefore they are at no risk of infection.

Groups 18 to 21 are key populations and their risk of infection is calculated as a function of HIV prevalence ($\rho_{i,r}$) and duration of exposure to the risk practice ($\delta_{18\text{-}21,r}$).

$$\lambda_{1,\mathrm{r}} = \xi_s \cdot \varphi + \xi_e \cdot \varphi \qquad \text{...38}$$

$$\lambda_{2,\mathrm{r}} = \xi_s \cdot \varphi \cdot o + \xi_e \cdot \varphi \qquad \text{...39}$$

$$\lambda_{3,\mathrm{r}} = \xi_s + \xi_e \qquad \text{...40}$$

$$\lambda_{4,\mathrm{r}} = \xi_s \cdot o + \xi_e \qquad \text{...41}$$

$$\lambda_{5,\mathrm{r}} = \xi_s + \xi_e \qquad \text{...42}$$

$$\lambda_{6,\mathrm{r}} = \xi_s \cdot o + \xi_e \qquad \text{...43}$$

$$\lambda_{7,\mathrm{r}} = \xi_e \cdot \left(\varphi \cdot \left(M_{1,r} \big/ \left(M_{1,r} + W_{1,r}\right) \right) + \left(W_{1,r} \big/ \left(M_{1,r} + W_{1,r}\right) \right) \right) \qquad \text{...44}$$

$$\lambda_{8,\mathrm{r}} = \xi_e \qquad \text{...45}$$

$$\lambda_{9,\mathrm{r}} = 0 \qquad \text{...46}$$

$$\lambda_{10,\mathrm{r}} = \rho_{10,\mathrm{r}} \cdot 1/\,\delta_{10,\mathrm{r}} \cdot \varphi \qquad \text{...47}$$

$$\lambda_{11,\mathrm{r}} = \rho_{11,\mathrm{r}} \cdot 1/\,\delta_{11,\mathrm{r}} \qquad \text{...48}$$

$$\lambda_{12,\mathrm{r}} = \rho_{12,\mathrm{r}} \cdot 1/\,\delta_{12,\mathrm{r}} \qquad \text{...49}$$

$$\lambda_{13,\mathrm{r}} = \left(\xi_s + \xi_e\right) \cdot \varsigma_{13} \cdot \varphi \qquad \text{...50}$$

$$\lambda_{14,\mathrm{r}} = \left(\xi_s + \xi_e\right) \cdot \varsigma_{14} \qquad \text{...51}$$

$$\lambda_{15,\mathrm{r}} = \left(\xi_s + \xi_e\right) \cdot \varsigma_{15} \qquad \text{...52}$$

$$\lambda_{16,\mathrm{r}} = 0 \qquad \text{...53}$$

$$\lambda_{17,\mathrm{r}} = 0 \qquad \text{...54}$$

$$\lambda_{18,\mathrm{r}} = \rho_{18,\mathrm{r}} \cdot 1/\,\delta_{18,\mathrm{r}} \qquad \text{...55}$$

$$\lambda_{19,\mathrm{r}} = \rho_{19,\mathrm{r}} \cdot 1/\,\delta_{19,\mathrm{r}} \qquad \text{...56}$$

$$\lambda_{20,\mathrm{r}} = \rho_{20,\mathrm{r}} \cdot 1/\,\delta_{20,\mathrm{r}} \qquad \text{...57}$$

$$\lambda_{21,\mathrm{r}} = \rho_{21,\mathrm{r}} \cdot 1/\,\delta_{21,\mathrm{r}} \qquad \text{...58}$$

At time zero the population in each group and region is divided between HIV susceptible $S_{t=0,i,r}$ and infected $Y_{t=0,i,r}$ individuals according to equations 59 and 60 where $\rho_{i,r}$ is the HIV prevalence in each group and province, $\kappa_{i,r}$ is the distribution of the population by group and province and A_r is the total adult (15-49) population by province.

$$S_{t=0,i,r} = (1 - \rho_{i,r}) \cdot \kappa_{i,r} \cdot A_r \quad \text{...59}$$

$$Y_{t=0,i,r} = \rho_{i,r} \cdot \kappa_{i,r} \cdot A_r \quad \text{...60}$$

The proportion of new infection per group in each province $\Phi_{i,r}$ is calculated as the number of new infections in each group and province, $\Omega_{i,r}$, over the total number of new infections in the province as shown in equations 61 and 62.

$$\Omega_{i,r} = \lambda_{i,r} \cdot S_{t=0,i,r} \quad \text{...61}$$

$$\Phi_{i,r} = \Omega_{i,r} / \sum_{i=1}^{21} \Omega_{i,r} \quad \text{...62}$$

In the interest of clarity, these are then aggregated into larger groups, better identifiable at the programmatic level as described in equations 63 to 71:

$$\Phi_{Unions,\ r} = \sum_{i=1}^{9} \Phi_{i,r} \quad \text{...63}$$

$$\Phi_{NeverMarriedMen,\ r} = \sum_{i=10}^{11} \Phi_{i,r} \quad \text{...64}$$

$$\Phi_{NeverMarriedWomen,\ r} = \Phi_{12,r} \quad \text{...65}$$

$$\Phi_{PreviouslyMarriedMen,\ r} = \sum_{i=13}^{14} \Phi_{i,r} \quad \text{...66}$$

$$\Phi_{PreviouslyMarriedWomen,\ r} = \Phi_{15,r} \quad \text{...67}$$

$$\Phi_{FSW,\ r} = \Phi_{18,r} \quad \text{...68}$$

$$\Phi_{FWID,\ r} = \Phi_{19,r} \quad \text{...69}$$

$$\Phi_{MSM,\ r} = \Phi_{20,r} \quad \text{...70}$$

$$\Phi_{MWID,\ r} = \Phi_{21,r} \quad \text{...71}$$

1.3. MODEL FITTING

A Bayesian approach, whereby prior knowledge on the processes investigated is taken into account in the analysis, was implemented using Metropolis, an MCMC algorithm. Prior information on demography, HIV prevalence, duration of sexual activity or risk practices and incidence patterns from the sub-Saharan African region is used to parameterise the model which is then fit to data from a specific setting (province). As described in the main text, prior means were calculated as the weighted average of the values obtained from the DHS survey of 19 Sub-Saharan countries (S2 Table) and the variance was obtained from the total sample size divided by 500 to give less weight to the priors (Table 3 in the main text).

The Metropolis algorithm was implemented to sample from the joint posterior distribution using a sequential updating tuned during the burnin period to reach an acceptance rate between 20% and 40% in order to improve efficiency. The Metropolis algorithm was implemented over 3 chains of 25,000 iterations each. The proposal distribution was a normal distribution with mean θ_{i-1} and a fixed variance for each model parameter.

The logarithm of the posterior probability p(θ|y) was calculated as the sum of the logarithms of the likelihoods p(y|θ) and of the prior probabilities p(θ) to an additive constant.

The log-likelihood functions were calculated as the log-probability of the data given the model and its parameters for the following data: the proportion of people in each group, the HIV prevalence in each group, the duration of sexual activity among never married men and women and the **total** number of new infections predicted to occur in the next year using the UNAIDS estimate as a proxy for data. The logarithm of the probability density estimate obtained for each data point given the model parameters was calculated using multinomial, binomial, normal and poisson probability density functions accordingly as shown in equations 72 to 75.

Poisson distributed outputs:

$$\ln\left(\pi\left(D_r \middle| I_r\right)\right) = -I_r + D_r \ln\left(I_r\right) - \ln\left(D_r!\right) \quad ...72$$

Where I_r is the model estimates of the number of new infections in province r, D_r is the number of new infections in province r as estimated by UNAIDS. If estimates are not available per province the total number of new infections is used instead.

Binomially distributed outputs:

$$\ln\left(\pi\left(\frac{Y_{i,r}}{N_{i,r}} \middle| \rho_{i,r}\right)\right) = \ln\left(N_{i,r}!\right) - \ln\left(Y_{i,r}!\right) - \ln\left(\left[N_{i,r} - Y_{i,r}\right]!\right) + Y_{i,r}\cdot\ln\left(p_{i,r}\right) + \left(N_{i,r} - Y_{i,r}\right)\cdot\ln\left(1 - p_{i,r}\right) \quad ...73$$

Where $N_{i,r}$ is the total number of people in the group in that province, $Y_{i,r}$ is the number of HIV positive people in the group in that province and $p_{i,r}$ is the HIV prevalence in the model for the group in that province.

Lognormally distributed outputs:

$$\ln\left(\pi\left(x_r \middle| \mu_r, \sigma\right)\right) = -\ln\left(x_r\right) - \frac{1}{2}\cdot\ln\left(2\pi\right) - \frac{1}{2}\cdot\ln\sigma^2 - \frac{1}{2\sigma^2}\cdot\left(\ln\left(x_r\right) - \mu_r\right)^2 \quad ...74$$

Where x_r is the observed mean value of duration of sexual activity among never married men and women in the data, μ_r is the model estimate for the mean duration of sexual activity and σ^2 is its variance which is assumed to be equal to the variance of the prior.

Multinomially distributed outputs:

$$\ln\left(\pi\left(N_{1,r}, N_{2,r}, ..., N_{n,r} \mid \rho_{1,r}, \rho_{2,r}, ..., \rho_{n,r}\right)\right) = \ln\left(n!\right) - \sum_{i=1}^{n}\ln\left(N_{i,r}!\right) + \sum_{i=1}^{n}\left(N_{i,r}\cdot\ln\left(\rho_{i,r}\right)\right) \quad ...75$$

Where $N_{i,r}$ is the number of individuals in group i and province r, n is the total number of groups for either men, women or unions and $p_{i,r}$ is the model estimate for the proportion of individuals in that group.

The logarithm of the posterior probability π(θ|D) is calculated as the sum of the logarithms of the prior probability π(θ) and the likelihood π(D|θ) (to an additional constant) as shown in equation 76 below.

$$\ln\left(\pi\left(\theta \middle| D\right)\right) \propto \sum_{i,r}\ln\left(\pi\left(D_{i,r} \middle| \theta_{i,r}\right)\right) + \sum_{i,r}\ln\left(\pi\left(\theta_{i,r}\right)\right) \quad ...76$$

Where $D_{i,r}$ is the data for group i in province r and $\theta_{i,r.}$ is the corresponding model parameter

2. MODEL VALIDATION

2.1. DESCRIPTION OF ALPHA NETWORK STUDIES

MANICALAND

The Manicaland cohort in Zimbabwe is an HIV/STD Prevention research initiative that has been underway in rural areas of eastern Zimbabwe since the early 1990s. Five surveys have been implemented among an open cohort in 12 study sites since 1998. Data on unions started being collected in the third survey so we limited our analysis to surveys 3 to 5. Ethical approval was obtained from the Imperial College Research Ethics Committee (ICREC_9_3_13), the Biomedical Research and Training Institute's IRB (AP91/10), and the Medical Research Council of Zimbabwe (MRCZ/A/681).

KARONGA

The Karonga prevention study in Malawi has been collecting demographic data since 2002 and HIV data since 2007. Four surveys including serological data have been collected so far of which two overlapped with sexual behaviour surveys and therefore provided sufficient data to carry out the validation. Ethical approval was obtained from the Malawi National Health Sciences Research Committee (#419).

RAKAI

The Rakai project started collecting cohort data in Uganda in 1988 and has allowed exploring fundamental questions on the virus' dynamics as well as on determinants of transmission including other STIs and circumcision. 14 surveys have been completed so far, of which four (surveys 11 to 14) were used for the validation. Ethical approval was obtained from the Uganda Virus Research Institute's Research and Ethics Committee and the Uganda National Council for Science and Technology.

KISESA

The Kisesa cohort has been providing data on HIV prevalence and sexual behaviours through seven surveys, demographic surveys are carried out separately during biannual household visits. Two of these surveys (five and six) provided suitable data for this analysis. The protocol was approved by the Lake Zone Institutional Review Board and the National Ethical Review Committee.

Written informed consent was a requirement for participation in each of the four studies, thumbprint in front of a witness was required for participants who could not write.

2.2. DATA ANALYSIS OF INCIDENCE BY RISK DETERMINANTS

To obtain the distribution of new infections by risk determinants between two subsequent surveys (defined as "rounds") in the four ALPHA sites studied, the population was disaggregated by marital and circumcision status: sexual activity in the past 12 months was determined through the date at last sex, marriage/cohabitation was defined as having been in a relationship for 6 months or more, never been married and previously been married was defined as never or having been in a relationship for 6 months or more in the past respectively and circumcision was self-reported. The distribution by sero-concordance status in unions was obtained from the linked unions' data where men and women were matched if they reported each other as current married/cohabiting partners.

As participants can change marital and circumcision status in the time period between two surveys, they were allocated to the group in which they spent the longest time if data was available to determine this (duration of marriage or duration of sexual activity for those who married or became sexually active between the two surveys respectively) or allocated randomly to either of the two groups otherwise. Sero-conversions were assumed to have occurred at the mid-point between the two surveys. All participants who seroconverted and

reported not being sexually active at one of the two surveys were allocated to the other group. Those who seroconverted and reported not being sexually active at both surveys were allocated to the never married or previously married groups based on whether they reported having been married/cohabited in the past. The unions' analysis was restricted to unions that were linked in both surveys (i.e. that remained together through the period between the two surveys).

The distribution of new infections by group defined by gender, marital status and circumcision status for each of the rounds in the four ALPHA sites is given in Tables S3 to S9. As women were over-represented in the surveys and only a proportion of unions were linked, the number of sero-conversions were rescaled to reflect an equal proportion of men and women in the population and to reflect the true proportion of married men and women in the linked unions' data. To implement corrections to the number of sero-conversions among each type of unions, the rescaled total number of new infections among married men and women was distributed according to the distribution of new infections observed among linked unions. The mean and variance of the duration of sexual activity among never men and women are also provided in these tables. Estimates of ART coverage among HIV positive people are shown in the tables; however, they were only directly obtained from this data analysis for Rakai. Estimates for the other sites were obtained from a published study of ART access in Alpha network sites[1].

2.3. Implementation of model validation

The model was fit to the total number of new infections summed across all sites to evaluate the model's ability to estimate the distribution of new infections by group in each site as well as by site (geographical distribution). Because the intervals between surveys (so within one round) were different across sites, the number of new infections in each site was rescaled assuming a 2 year interval between surveys before summing across them. The original priors obtained from the DHS meta-analysis and literature reviews were used and only information on the population distribution, prevalence by group, duration of sexual activity and total number of new infections was included in the calculation of the likelihood (i.e. no information on the number of new infections by group or site was included). The trace plots of the 3 chains of the MCMC are shown in S2 Fig and S3 Fig for each of the fitted parameters. The incidence parameters are shown in S2 Fig and were constant across sites while the other parameters (S3 Fig) varied by site and round. All chains reached convergence within the first 2000 iterations and mixed well.

3. Application in countries

The model was applied to 6 countries in the region: Gabon, Kenya, Malawi, Rwanda, Swaziland and Zambia. Data on the distribution and HIV prevalence by marital status, and circumcision status as well as on the average duration of sexual activity was extracted from the DHS for each province using appropriate DHS survey weights that adjust for biases arising from the survey design which oversamples certain groups and certain locations. The raw sample size of each of the estimates extracted was also obtained as it is used in the calculation of the log-likelihood in the Metropolis algorithm. Information of the size and HIV prevalence among key populations was also obtained from a review of the peer reviewed literature, the US Census Bureau HIV database, government surveys and reports to international organisation such as the UNAIDS country profiles and UNGASS reports. The estimates and corresponding sample sizes for each province in each country are provided in S10 Table and S11 Table respectively. Data on key populations was mostly missing and estimates of the size of key populations were often obtained using mixed methods or experts' opinion so no estimate of the sample size was available. In these cases the sample size was arbitrarily set at 100 to reflect important uncertainty around them.

S4 Fig provides a graphical representation of the distribution of the population by province and by group in each province as estimated by the IPM as part of the process to estimate the distribution of new infections acquired in each group and province shown in Figure 6 of the main text. S5 Fig, in turn describes the distribution of the HIV prevalence burden by province and by group in each province as estimated by the IPM as part of this process. These facilitate the interpretation of the results shown in Figure 6 and 7 of the main text.

S12 Table provides a list of Sub-Saharan countries disaggregated by time of demographic and health survey to determine where the model can be applied based on data availability. 11 countries in the region (last 11 in the table) do not have a population survey with HIV testing and so would not be able to apply the model at this stage.

4. TRANSMISSION MODULE

Values for the number of new infections acquired per group and province and associated parameter values obtained from applying the MCMC algorithm to the acquisition model and resampling were directly used as inputs in the transmission module. The weight of transmission for each group j to the number of new infections in group i ($W_{i,j,r}$) was calculated based on the mixing matrix of i ($M_{i,j,r}$), the HIV prevalence in group j ($p_{j,r}$), the transmissibility of group j (T_j), the coverage of ART in group j (ζ_j) as shown in equation 77. The proportion of infections in group i transmitted by group j, $P_{i,j,r,}$ corresponds to this weight rescaled as shown in equation 78. Given that the proportion of new infections in unions arising from within partnership transmission is available from the acquisition model it is calculated in equation 79. The proportion of new infections among unions transmitted by other groups is rescaled accordingly in equation 80 and the values for the proportion of infections in unions arising from within partnership transmission are replaced in equation 81. The number of new infections $I_{i,j,r}$ in i transmitted by j corresponds to the proportion $P_{i,j,r}$ times the total number of new infections in group I as shown in equation 82. $\Gamma_{j,r}$ in equation 83 corresponds to the total number of new infections transmitted by group j across all groups and $\Pi_{j,r}$ in equation 84 corresponds to the total proportion of new infections which are transmitted by group j. This is calculated for each province r.

$$W_{i,j,r} = \mathrm{M}_{i,j,r} \cdot p_{j,r} \cdot \mathrm{T}_j \cdot \zeta_j \quad \text{...77}$$

$$\mathrm{P}_{i,j,r} = W_{i,j,r} \Big/ \sum_{j=1}^{21} W_{i,j,r} \quad \text{...78}$$

$$S_{i=1\to 6,\, j=i,r} = \xi_s \varphi_j \Big/ \left(\xi_s \varphi_j + \xi_e\right) \quad \text{...79}$$

$$\mathrm{P}_{i=1\to 6,\, j\neq i,r} = \left(1 - S_{i,j=i,r}\right)\left(\mathrm{P}_{i,j,r} \Big/ \left(1 - \mathrm{P}_{i,j=i,r}\right)\right) \quad \text{...80}$$

$$\text{...81}$$

$$\mathrm{P}_{i=1\to 6,\, j=i,r} = S_{i,j=i,r} \quad \text{...82}$$

$$\mathrm{I}_{i,j,r} = P_{i,j,r}\,\Omega_{i,r}$$

$$\Gamma_{j,r} = \sum_{i=1}^{21} \mathrm{I}_{i,j,r} \quad \text{...83}$$

$$\Pi_{j,r} = \Gamma_{j,r} \Big/ \sum_{j=1}^{21} \Gamma_{j,r} \quad \text{...84}$$

The baseline transmissibility was set for married women at T_{1-2}=0.1 and it was assumed to be 2 fold higher among married men. It was assumed to be slightly lower among never-married women at T_{12}=0.08 because of higher condom use and 2 fold higher among never married men at T_{10-11}=0.16. The transmissibility for MSM was assumed to be 0.3 due to higher transmission during anal sex and the transmissibility among FSW was assumed to be equal than among married women due to higher prevalence of STIs but higher condom use. Among PWID it was assumed to be 2 times higher compared to married women due to the high prevalence of STIs. The relative transmissibility was assumed to be the same for all married women and for all married men respectively independently of the type of partnership based on sero-status. The mixing matrix parameters were assigned a beta prior distribution and were based on information on age-mixing and on the proportion reporting extramarital sex and paying for sex by marital status in the DHS. Trees are constructed so that the mixing matrix parameters for one group i ($M_{i,j,r}$) always sum to 1 across j when sampling from the prior distribution. The trees are defined by the parameters χ listed in S13 Table so that if individuals from a group i mix with five other groups j there will be four χ parameters defining the mixing matrix ($M_{i,j,r}$). We constrained the structure of the mixing matrix to reflect the following assumptions: MSM only mixed with other MSM, with MWID and with women as it is considered that all MSM are represented in that group with the exception of MWID for whom the main risk comes from sharing injecting equipment but who also have sex with men. MWID mix with other PWID, with MSM and with women in the population. FWID mix with other PWID and

with men in the population. All other groups exclusively mix with members of the opposite sex. Prior distributions are listed in S13 Table.

References

1. Wringe A, Floyd S, Kazooba P, et al. Antiretroviral therapy uptake and coverage in four HIV community cohort studies in sub-Saharan Africa. *Tropical Medicine & International Health* 2012; **17**(8): e38-e48.
